# An Artificial Intelligence Approach for Tackling Conformational Energy Uncertainties in Chiroptical Spectroscopies

**DOI:** 10.1002/anie.202307053

**Published:** 2023-07-10

**Authors:** Gabriel Marton, Mark A. J. Koenis, Hong-Bing Liu, Carole A. Bewley, Wybren Jan Buma, Valentin Paul Nicu

**Affiliations:** Provitam Foundation, Caisului Street 16, Cluj-Napoca, Romania; Van’t Hoff Institute for Molecular Sciences, University of Amsterdam, Science Park 904, 1098 XH Amsterdam, The Netherlands; Laboratory of Bioorganic Chemistry, National Institute of Diabetes and Digestive and Kidney Diseases, National Institutes of Health Bethesda, Maryland 20892-0820, United States; Laboratory of Bioorganic Chemistry, National Institute of Diabetes and Digestive and Kidney Diseases, National Institutes of Health Bethesda, Maryland 20892-0820, United States; Van’t Hoff Institute for Molecular Sciences, University of Amsterdam, Science Park 904, 1098 XH Amsterdam, The Netherlands; Radboud University, Institute for Molecules and Materials, FELIX Laboratory, Toernooiveld 7c, 6525 ED Nijmegen, The Netherlands.; Provitam Foundation, Caisului Street 16, Cluj-Napoca, Romania

**Keywords:** Density Functional Calculations, Absolute configuration, Chiroptical spectroscopies, Genetic algorithm, Hierarchical clustering

## Abstract

Determination of the absolute configuration of chiral molecules is a prerequisite for obtaining a fundamental understanding in any chirality-related field. The interaction with polarised light has proven to be a powerful means to determine this absolute configuration, but its application rests on the comparison between experimental and computed spectra for which the inherent uncertainty in conformational Boltzmann factors has proven to be extremely hard to tackle. Here we present a novel approach that overcomes this issue by combining a genetic algorithm that identifies the relevant conformers by accounting for the uncertainties in DFT relative energies, and a hierarchical clustering algorithm that analyses the trends in the spectra of the considered conformers and identifies on-the-fly when a given chiroptical technique is not able to make reliable predictions. The effectiveness of this approach is demonstrated by considering the challenging cases of papuamine and haliclonadiamine, two bis-indane natural products with eight chiral centres and considerable conformational heterogeneity that could not be assigned unambiguously with current approaches.

## Introduction

The absolute configuration (AC) of a chiral molecule is in many aspects key to its use and applications in chemical, biological and health sciences. The assignment of the AC is thus a critical step in a wide range of scientific areas ranging from purely fundamental research to commercial applications.^[1]^ For AC determinations of molecules under solvated conditions chiroptical spectroscopies are the current state-of-the-art.^[1–4]^ Although significant advancements have been achieved experimentally, the weakness of these techniques lies in the fact that determining the AC requires a comparison between an experimental spectrum and a simulated spectrum that at times may be less accurate. The simulated spectrum is normally obtained as a Boltzmann average of conformer spectra computed with, for example, Density Functional Theory (DFT) for the low-energy conformers within an energetic window of 2–3 kcal/mol. The reason for this is twofold. Firstly, the neglected high-energy conformers have very small populations at room temperature and do not contribute significantly to the Boltzmann-averaged simulated spectrum. Secondly, the DFT calculations can become very expensive if too many conformations are considered.

In the last two decades this approach has been tested extensively and successfully using electronic circular dichroism (ECD), vibrational circular dichroism (VCD) and Raman optical activity (ROA) spectroscopic techniques.^[1,3,4]^ While this represents an indisputable proof of how far computational chemistry and in particular DFT simulations have come, it is important to realise that the relative energies computed with DFT are not absolute quantities but are intrinsically associated with uncertainties^[5–8]^ that often are larger than 2–3 kcal/mol. The implicit assumption that the Boltzmann factors calculated with DFT are correct is thus a serious weakness of the current protocol. Compounding on this weakness is the observation that the difficulties associated with a confidential determination rise steeply as the conformational flexibility of the compound and the number of chiral centres increases. Unfortunately, it is especially in this direction that there is a steeply increasing need for stereochemical analyses.^[3,4]^

In the recent past we have started to address this shortcoming using a genetic algorithm that considered the calculated energies as having an intrinsic uncertainty.^[9]^ In the present work we propose a significantly more elaborated protocol that combines two artificial intelligence (AI) algorithms. Specifically, a hierarchical clustering algorithm and a more effective and efficient version of the recently introduced genetic algorithm (GA) VCD protocol, which now enables the simultaneous optimisation of more types of chiroptical spectra.

**Scheme 1.**
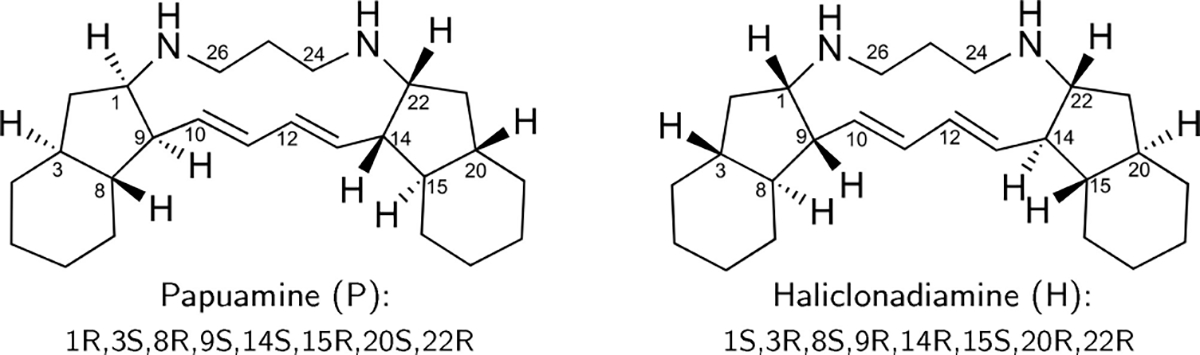
Schematic representation of the structures of Papuamine and Haliclonadiamine. The configuration of the two molecules is also indicated.

As a case study we consider papuamine (1) and haliclonadiamine (2), two bis-indane natural products isolated from the marine sponge *Haliclona* sp.. As shown in Scheme 1, these two compounds are very flexible, have very similar structures and share eight chiral centres. This makes them a difficult case to study with chiroptical spectroscopies. Indeed, initial studies^[10–14]^ on (1) and (2) reported that C_22_ is the only chiral centre that has an opposite configuration in the two compounds. However, a more recent study^[15]^ that combined X-ray crystallography and electronic circular dichroism (ECD) spectroscopy came to the conclusion that the opposite is actually true, i.e., haliclonadiamine has a 1*S*,3*R*,8*S*,9*R*,14*R*,15*S*,20*R*,22*R* configuration, while papuamine has a 1*R*,3*S*,8*R*,9*S*,14*S*,15*R*,20*S*,22*R* configuration. It should be noted, however, that in the X-ray crystallographic studies only one particular conformation is selected and this conformer was the only one considered when simulating the experimental ECD spectra. Therefore, the previous investigations could not determine whether these compounds have under actual biological (solvated) conditions more than one conformation populated at room temperature—a paramount detail for understanding their biological function. These complications make papuamine and haliclonadiamine ideal examples for illustrating the weakness of the standard protocol and the effectiveness of the newly proposed one.

We will show that the protocol proposed here avoids the need to rely on inaccurate Boltzmann factors. As a result, it is capable of providing reliable AC assignments and insight into conformational heterogeneities even in the difficult situations described above—flexible systems of considerable size featuring a large number of chiral centres. In addition, the new protocol also provides an unambiguous tool to identify instances in which a particular type of chiroptical spectroscopy may not be able to provide reliable AC assignments.

## Results and Discussion

**Figure 1.**
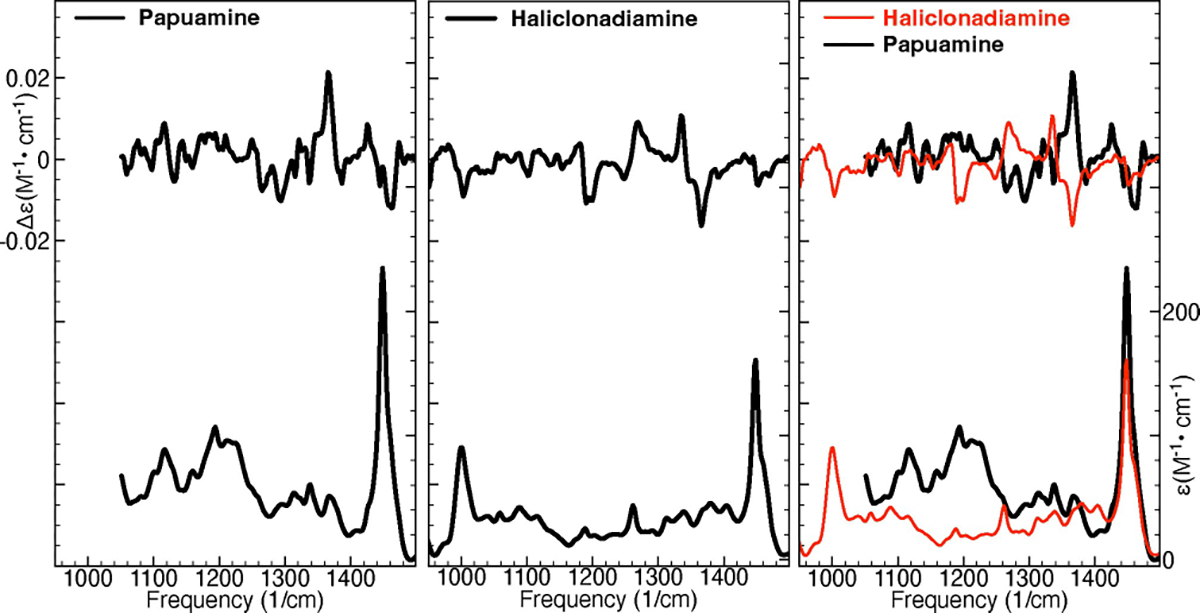
Experimental IR and VCD spectra of papuamine (left) and haliclonadiamine (middle). The right panel shows the two sets of experimental spectra overlayed.

Figure 1 shows the experimental IR and VCD spectra of papuamine and haliclonadiamine. As shown in the right panel of Figure 1, the two VCD spectra have roughly opposite signs between 1180 and 1400 cm^−1^. This is a consequence of the fact that 7 of the 8 chiral centres have an opposite configuration in the two compounds. Importantly, this allows us to conclude that VCD spectroscopy can unambiguously discern between papuamine and haliclonadiamine, in contrast to previous electronic circular dichroism (ECD) studies in which the intensity of basically one single ECD band with the same sign in both compounds was the leading criterion for distinguishing the two compounds.^[15]^

The DFT calculations predict that haliclonadiamine has 4 low-energy conformers within 1 kcal/mol and 6 within 2 kcal/mol, while papuamine has 4 conformers within 1 kcal/mol and 13 within 2 kcal/mol. These conformers account for 96% and 95% of the DFT Boltzmann-weighted spectra simulated for haliclonadiamine and papuamine, respectively.

**Figure 2.**
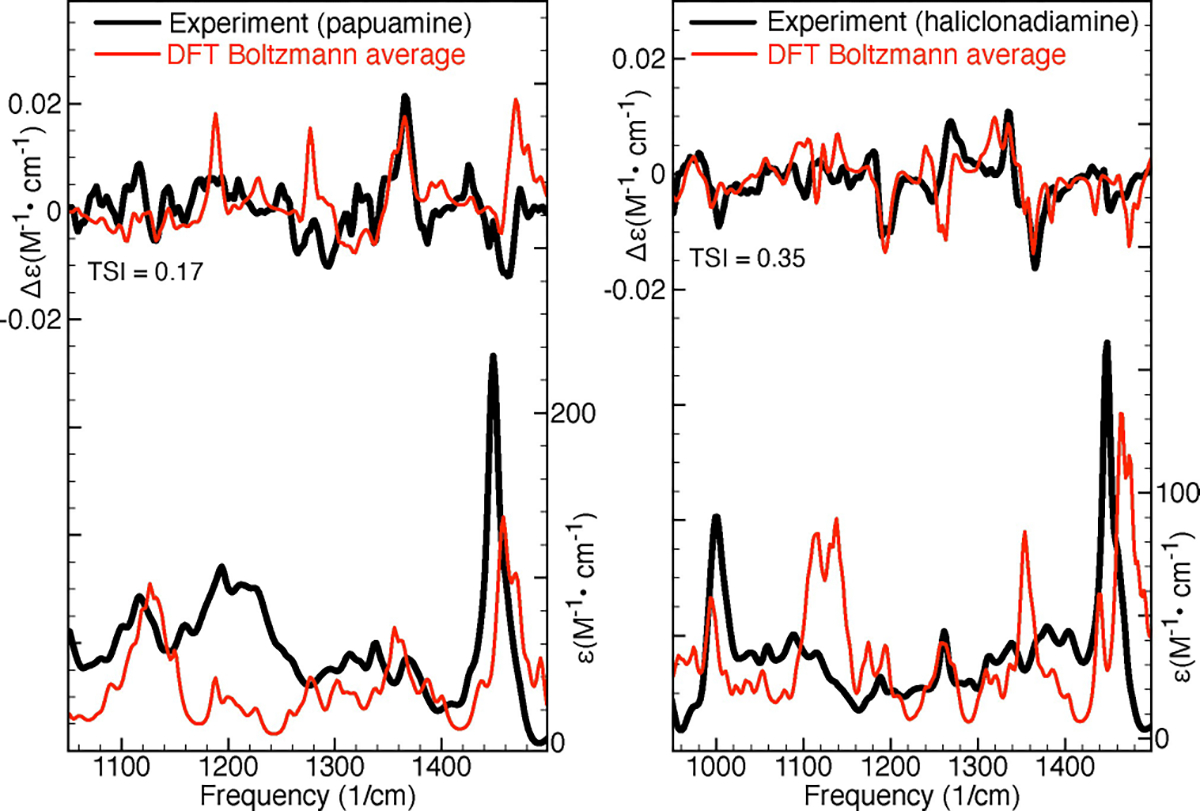
Comparison of experimental and simulated IR and VCD spectra of papuamine (left) and haliclonadiamine (right). The simulated spectra have been obtained by averaging the spectra computed for the considered conformers using the DFT Boltzmann factors. The Tanimoto similarity index (TSI) listed values were computed for the experimental and simulated VCD spectra in the frequency interval between 950 and 1500 cm^−1^

Figure 2 shows comparisons between the experimental IR and VCD spectra and the Boltzmann-averaged simulated spectra (obtained using the factors predicted by DFT). The Tanimoto similarity index (TSI), which is identical to the SymVCD index introduced by Shen et al.,^[16]^ will be used to quantify the similarity between the various types of experimental and simulated chiroptical spectra considered in this work. As can be seen in Figure 2, the TSI values computed between the experimental and simulated VCD spectra are rather low, 0.17 for papuamine and 0.35 for haliclonadiamine. While the agreement between calculations and experiment may seem satisfactory for haliclonadiamine—especially when compared to the case of papuamine—previous investigations^[16–19]^ have concluded that accurate AC assignments require SymVCD values larger than the 0.35 value obtained here.

**Figure 3.**
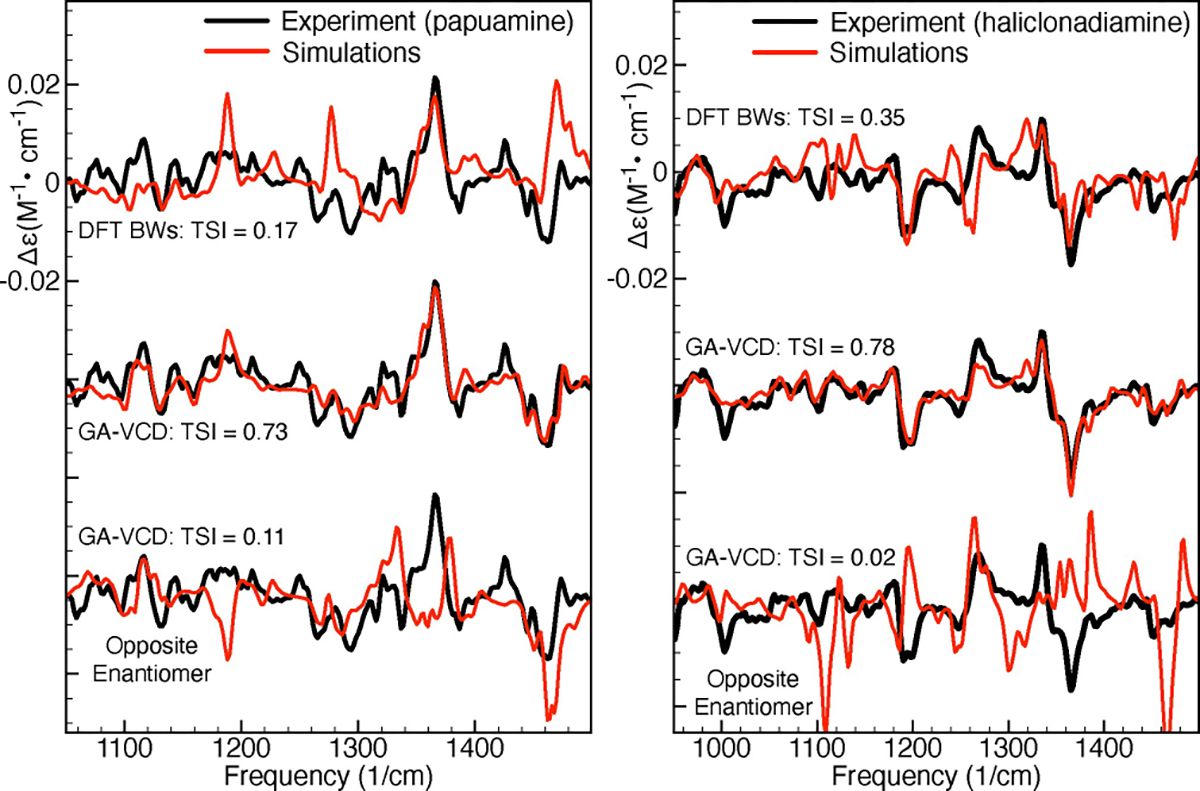
Application of the GA-VCD protocol to papuamine (left) and haliclonadiamine (right). The simulated spectra have been obtained by averaging the spectra computed for the considered conformers using: (1) the DFT Boltzmann factors (DFT BWs, upper comparison), (2) the Boltzmann factors obtained by fitting the experimental spectrum using the genetic algorithm (GA-BWs, middle comparison), and (3) the Boltzmann factors obtained by fitting the experimental spectrum using the genetic algorithm and the spectra of the computed conformers multiplied by minus one (GA-BWs, lower comparison). The TSI values were computed for the experimental and simulated VCD spectra in the frequency interval between 950 and 1500 cm^−1^.

Figure 3 as well as Figure S6 in the Supporting Information show the results obtained by fitting the experimental spectra using a genetic algorithm. For comparison, Figure 3 also shows the Boltzmann-averaged spectra based on the DFT calculated energies. As can be seen in the left panel, the GA-VCD spectrum obtained using the calculations for the 1*R*,3*S*,8*R*,9*S*,14*S*,15*R*,20*S*,22*R* configuration reproduces very well the experimental spectrum of papuamine (TSI 0.73), while the GA-VCD spectrum obtained for the opposite enantiomer fails to do so (TSI 0.11). We can thus unambiguously assign the absolute configuration of the experimental papuamine sample as 1*R*,3*S*,8*R*,9*S*,14*S*,15*R*,20*S*,22*R.* Important to notice is that the DFT Boltzmann-weighted spectrum of papuamine (TSI 0.16) does not reproduce the experiment significantly better than the GA-VCD spectrum associated with the opposite papuamine enantiomer (TSI 0.11). It is therefore clear that it is not possible to make an unambiguous AC assignment for papuamine using the Boltzmann-weighted spectra.

For haliclonadiamine, the standard DFT Boltzmann averaged spectrum has a TSI value of 0.35, while the GA-VCD spectra associated with the 1*S*,3*R*,8*S*,9*R*,14*R*,15*S*,20*R*,22*R* configuration and its enantiomer have TSI values of 0.78 and 0.02, respectively. Thus, the absolute configuration of the haliclonadiamine sample is 1*S*,3*R*,8*S*,9*R*,14*R*,15*S*,20*R*,22*R.* We therefore conclude that the present analysis performed under solution conditions confirms the recent reassignment^[15]^ of the haliclonadiamine configuration that was based on X-ray diffraction studies.

It is instructive to examine how the Tanimoto similarity index, calculated between experimental VCD spectra and GA-VCD simulated spectra, depends on the energy threshold (Δ*E*) used in the genetic algorithm—Δ*E* representing the uncertainty of relative energies computed using DFT. The first two columns of Table 1 display the TSI values obtained from the calculations performed for the correct configurations of haliclonadiamine and papuamine. In the case of papuamine, the overlap increases quickly from 0.17 at Δ*E* = 0.0 kcal/mol (equivalent to the DFT Boltzmann-weighted spectrum) to 0.45 at Δ*E* = 1.0 kcal/mol to 0.59 at Δ*E* = 3.0 kcal/mol and finally to 0.73 at Δ*E* = 5.0 kcal/mol. A further increase of ΔE further from 6 to 10 kcal/mol does not bring a significant increase in the similarity index, which remains 0.74. No significant changes are observed in the relevant GA-VCD conformers and their populations after Δ*E* = 5 kcal/mol (see Figure S7 in the SI). The same trends are observed for haliclonadiamine for which slightly larger overlaps are obtained for a given Δ*E* value than for papuamine. It can therefore be concluded that once all relevant conformers are present in the energetic window in which the genetic algorithm is allowed to search, an optimum and unique set of Boltzmann factors is found very quickly.

Further insight is obtained from Table 2 where we compare predictions made for individual haliclonadiamine conformers using the DFT relative energies and the GA-VCD protocol (see Table S-VII in the Supporting Information for the equivalent papuamine analysis). As can be seen in the upper section of Table 2, the conformers predicted by DFT to be relevant have small Tanimoto indices (0.25 or lower). On the other hand, the GA-VCD conformers, given in the lower section of Table 2, have significantly larger Tanimoto indices. This shows that the genetic algorithm does not combine the spectra of the various conformers blindly. Regardless of how many conformers one has considered, the algorithm takes into account only the conformers that it has identified to have a higher degree of similarity with the experimental spectrum.

**Figure 4.**
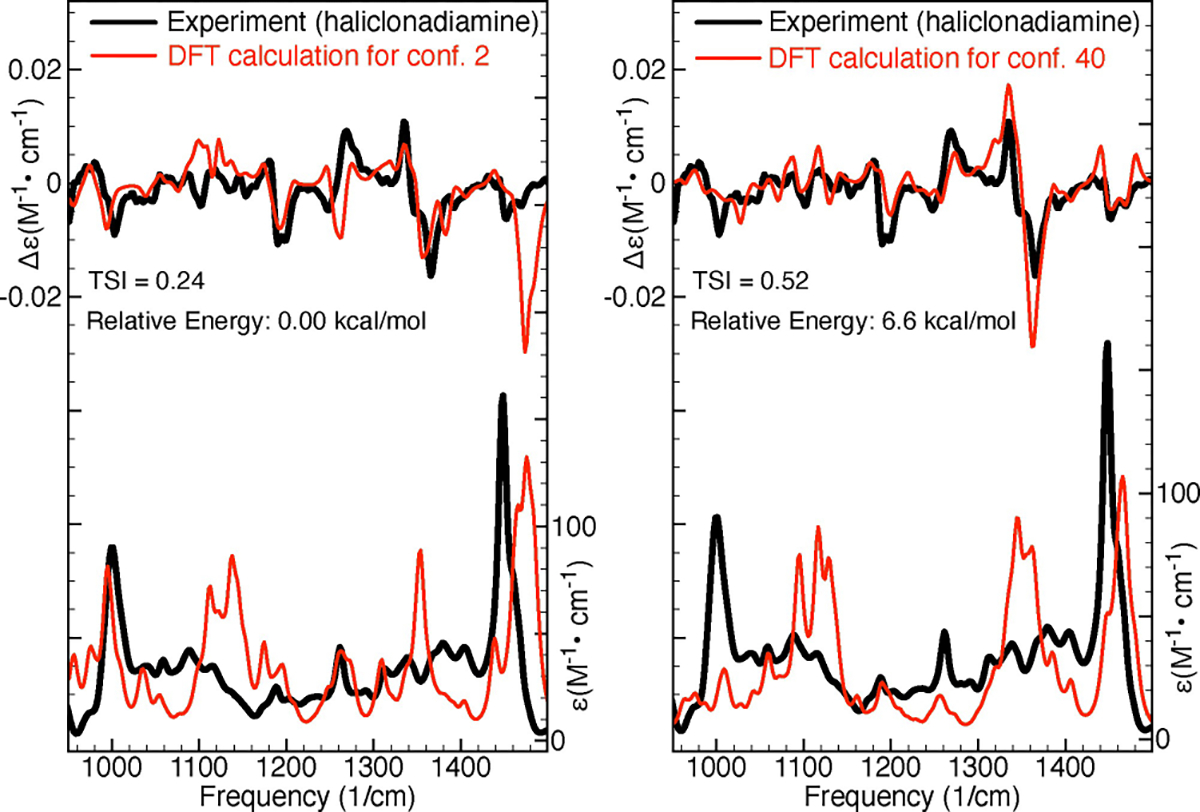
Comparison of experimental IR and VCD spectra of Haliclonadiamine and the spectra computed for the conformers 2 and 40 of Haliclonadiamine. The TSI values were computed for the experimental and simulated VCD spectra in the frequency interval between 950 and 1500 cm^−1^.

Besides large TSI values the GA-VCD conformers are also characterised by Δ*E* values that are significantly larger than those of the DFT conformers (see Table 2 here and Table S-VII in the SI). To showcase this, we will have a closer look at conformers 2 and 40 of Haliclonadiamine. Conformer 2, which was predicted by DFT to be the most important conformer, has a DFT Boltzmann population of 37% and a TSI of 0.24. Conformer 40, on the other hand, was identified by the GA-VCD protocol as the dominant conformer and has a GA-VCD Boltzmann population of 19% and a TSI of 0.52. Figure 4 compares the experimental IR and VCD spectra with the spectra computed for these two conformers. As can be seen, for both conformers a fair agreement with the experimental IR spectra is obtained. On the other hand, when comparing the VCD spectra we find that the spectrum of conformer 40 reproduces the experiment much better than the spectrum of conformer 2. This has two major consequences: (1) it increases confidence in the predictions made by the genetic algorithm and (2) it raises doubts about the predictions made by DFT for the Boltzmann factors. Essentially, if the experimental VCD spectrum is taken as reference, then it is reasonable to conclude that at room temperature, conformer 40 has a significantly higher population than conformer 2. This conclusion, however, is at odds with the predictions made by the vacuum DFT calculations, which found conformer 40 to have an energy that is 6.6 kcal/mol higher than that of conformer 2. This situation is observed also for the other DFT and GA-VCD conformers listed in Table 2. It is therefore clear that most conformers identified by GA-VCD as important, have relatively high DFT energies and large TSI values.

In an attempt to obtain relative energies that align better with the experimental observations, calculations were carried out for conformers 2 and 40 at various other levels of theory. As summarized in Table 3, these additional calculations did not reverse the energetic ordering of conformers 2 and 40. They only predicted a lower energetic gap between the two conformers. The vacuum BP86/TZP and B3LYP/TZP calculations estimate a relative energy larger than 6 kcal/mol, while the same levels of theory in combination with the COSMO continuum model yields values between 4.1 and 4.6 kcal/mol. The BP86/TZP/COSMO DFTD calculation, which also accounts for the London dispersion interactions, predicts the smallest energetic gap, i.e, 3.7 kcal/mol which is still too high to lead to a meaningful population for conformer 40 at room temperature. To further validate these results, B3LYP/TZP calculations were performed for all haliclonadiamine and papuamine conformers (i.e., 146 and 134 conformers, respectively). Since no significant changes were observed, we can conclude that for the two molecules considered here, the levels of theory that are typically employed for computing DFT VCD spectra are not capable of producing accurate Boltzmann factors.

The good agreement between the experimental and simulated spectra in Figures 3 and 4 assures us that the GA-VCD conformations are populated at room temperature. This enables us to estimate the uncertainty in the calculated relative DFT energies. The data in the fourth column of the lower section of Table 2 show that DFT predicts for the dominant GA-VCD conformers high relative energies (up to 10 kcal/mol). On the other hand, no further improvement in the agreement between the GA-VCD spectrum and the experiment is observed for Δ*E* values larger than 5 kcal/mol (see also Figure S7). This suggests an uncertainty for the DFT relative energies of at least 5 kcal/mol.

**Figure 5.**
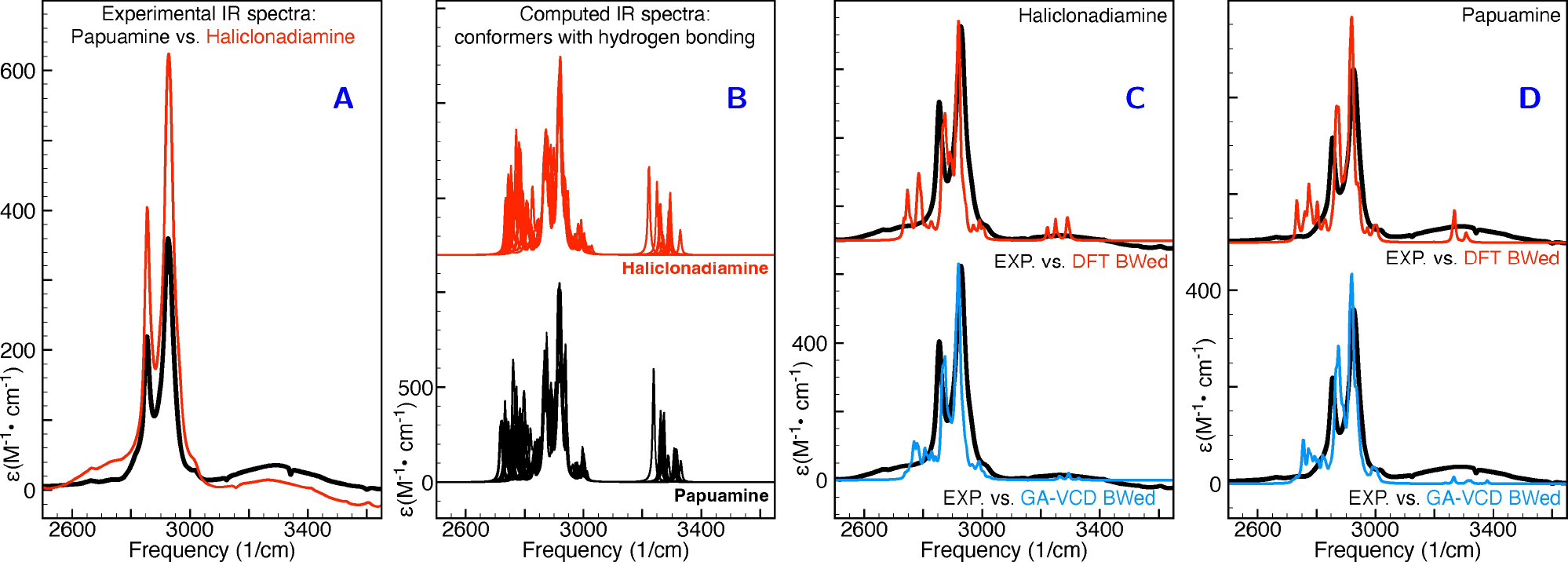
Comparison of experimental and simulated VA spectra in the CH and NH stretching region. Panel A: comparison of experimental VA spectra of haliclonadiamine and papuamine. Panel B: comparison of the spectra simulated for all conformers listed in Table 2. Panels C and D: comparison between experiment and simulated spectra of haliclonadiamine (C) and papuamine (D). The simulated spectra in panels C and D were obtained by averaging the spectra of the individual conformers using the DFT Boltzmann factors (DFT BWed) and the GA-VCD weights (GA-VCD BWed) listed in Table 2 and in Table S1 in the SI.

Interestingly, we find that the low-energy DFT conformers are characterised by a weak intramolecular hydrogen bond between the two NH groups, while in the conformers that according to the genetic algorithm fits are dominantly contributing to the VCD spectrum of haliclonadiamine the NH groups are directed in such a way that such a hydrogen bond is not possible (see Table 2 as well as Table S-VII in the SI). Inspection of the NH stretch region in the VA spectrum of haliclonadiamine reveals a broad band around 3270 cm^−1^ (see Figure 5). The broadness of this band might in first instance be taken as an indication of hydrogen bonding, but further inspection of the VA spectra calculated for the various conformers reveals that their NH stretch frequencies span the same range as covered by the experimentally observed band. In combination with the low intensity of the band in the experimental spectrum this observation strongly suggests that indeed conformations with no or quite weak intramolecular N–H···N–H bonds are dominantly present under the employed solution conditions. In the case of papuamine, the situation is less clear-cut. Here the VA spectrum shows a broad band as well, but we strongly suspect that in this case the VA spectrum may have suffered from the presence of minute amounts of water. We therefore hesitate to draw any conclusions from this part of the spectrum. Further support for our conclusions comes from the X-ray structure reported for the two compounds.^[15]^ Although these structures have been obtained for the the trifluoroacetic salt in which the two NH groups are protonated, it is interesting to notice that the conformation of haliclonadiamine as it is present in the crystal is also an extended one in which intramolecular hydrogen bonds would not be possible.

**Figure 6.**
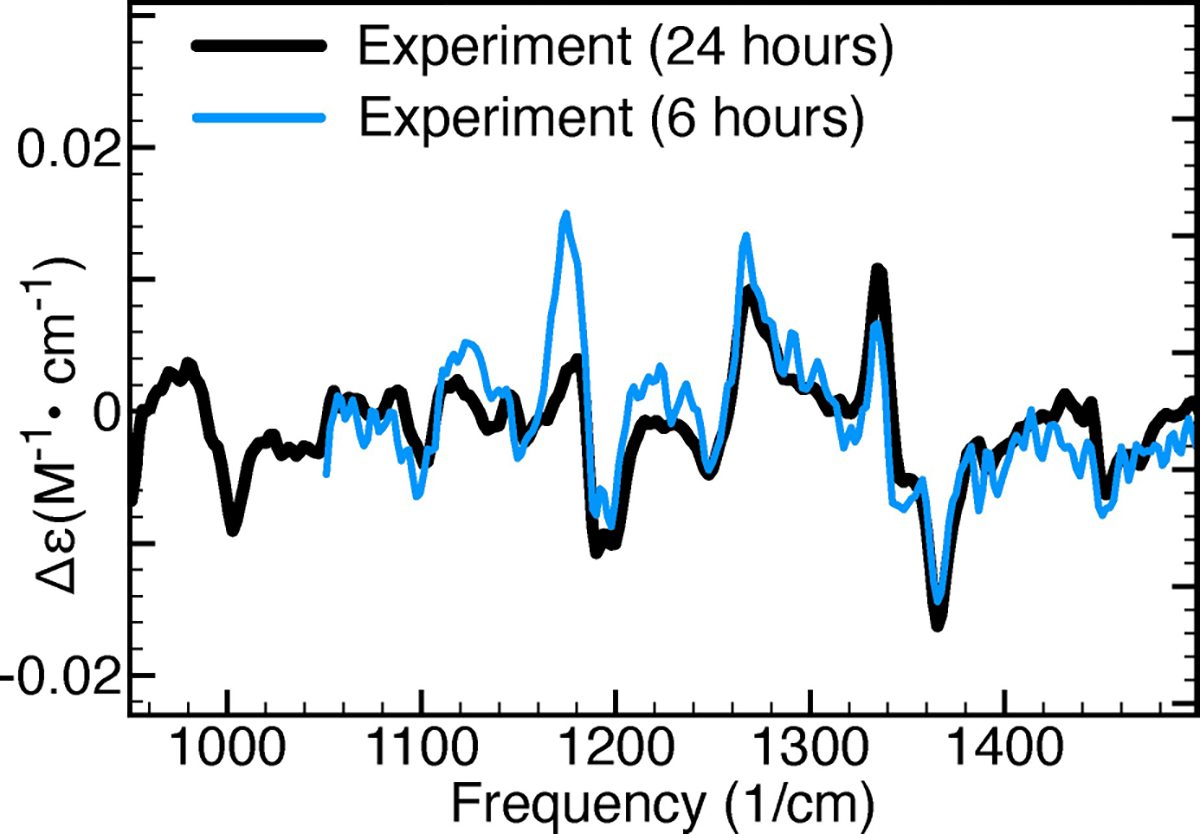
Comparison of two sets of experimental VCD spectra. The black spectrum was measured for 24 hours, while the blue spectrum for 6 hours.

Our next objective is to evaluate how the predictions made by the GA-VCD protocol are influenced by the levels of noise in the experimental VCD spectrum. This question holds significant importance as the study of natural products frequently encounters situations where access to both enantiomers and/or abundant sample quantities is limited, which often reduces the signal-to-noise ratios in the VCD spectra. To this end, we consider in Figure 6 two separate VCD measurements made for haliclonadiamine during a period of 6 and 24 hours, respectively. Table 4 reports the results obtained when simulating these spectra using the genetic algorithm. Firstly, we note that a lower signal-to-noise leads to significantly smaller TSI values which indicates that the genetic algorithm is unable to simulate effectively experimental artefacts. Secondly, it is seen that for both spectra the genetic algorithm predicts to a major extent the same conformers to be populated. These are two important observations because they imply that experimental artefacts are not prone to lead to incorrect conclusions on the relevance of conformers (see Table S-VI in the SI). At the same time, it is noticed that the GA Boltzmann factors associated with the two experimental spectra show clear differences. Thus, although qualitatively the two sets of experimental spectra give rise to the same conclusions, quantitatively the obtained results do differ.

It should therefore be acknowledged that—although the GA-VCD protocol yields a substantially higher conformer resolution than the conventional DFT approach—the predicted GA Boltzmann factors may still possess considerable uncertainties. For this reason, we have also investigated the dependence of the GA Boltzmann factors on (i) normal mode scaling factors and (ii) the fitness functions used by the genetic algorithm (see Section 3 of the SI). This additional study has shown clearly that all combinations of scaling factors and fitness functions leading to high TSI values consistently identify the same set of relevant conformers. It can thus be concluded that the GA approach yields consistent and robust results.

Further, we would like to asses the reliability of the predictions made using the genetic algorithm. We start by noting that the Tanimoto similarity values obtained when fitting the experimental VCD spectra using the calculations performed for the opposite enantiomers reflect the reliability of the AC predictions made using the genetic algorithm. As can be seen in the third and fourth columns in Table 1, the largest value obtained for papuamine is +0.114, while for haliclonadiamine it is +0.019. In practice, this means that for an uncertainty in the computed DFT relative energies of up to 10 kcal/mol, the experimental VCD spectra of papuamine and haliclonadiamine cannot accidentally be reproduced by calculations performed for their enantiomers. We thus conclude that the AC assignment on the basis of the GA-VCD protocol is very reliable.

**Figure 7.**
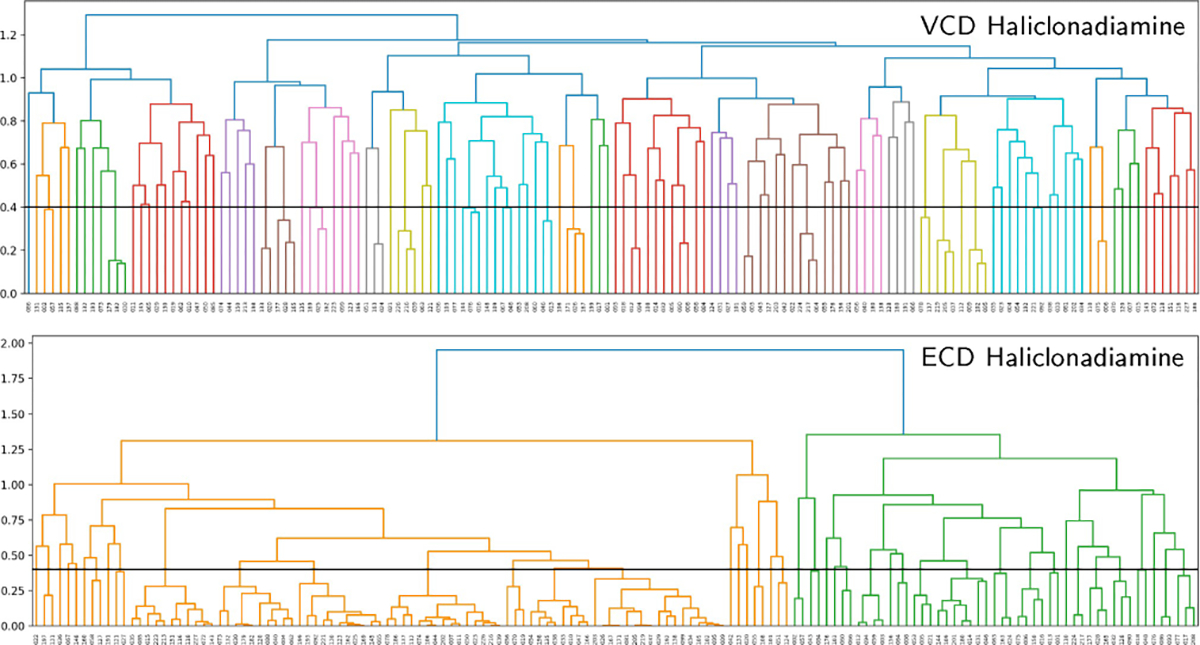
Dendrograms showing how the haliclonadiamine conformers are grouped into families according to the similarity of their VCD and ECD spectra.

**Figure 8.**
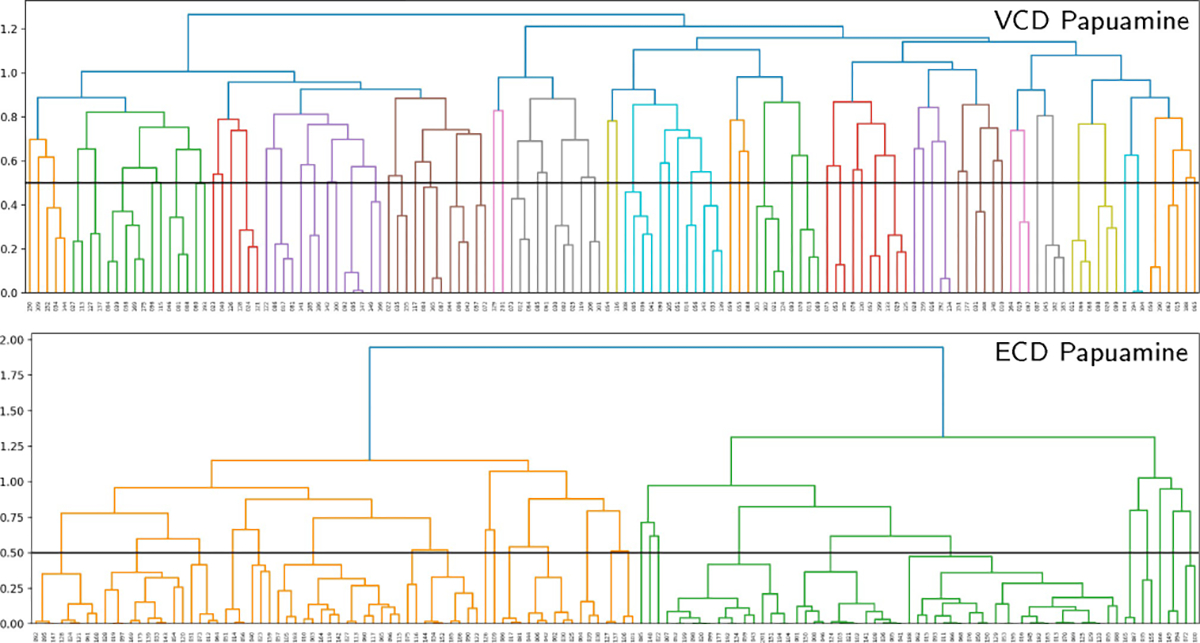
Dendrograms showing how the papuamine conformers are grouped into families according to the similarity of their VCD and ECD spectra.

**Figure 9.**
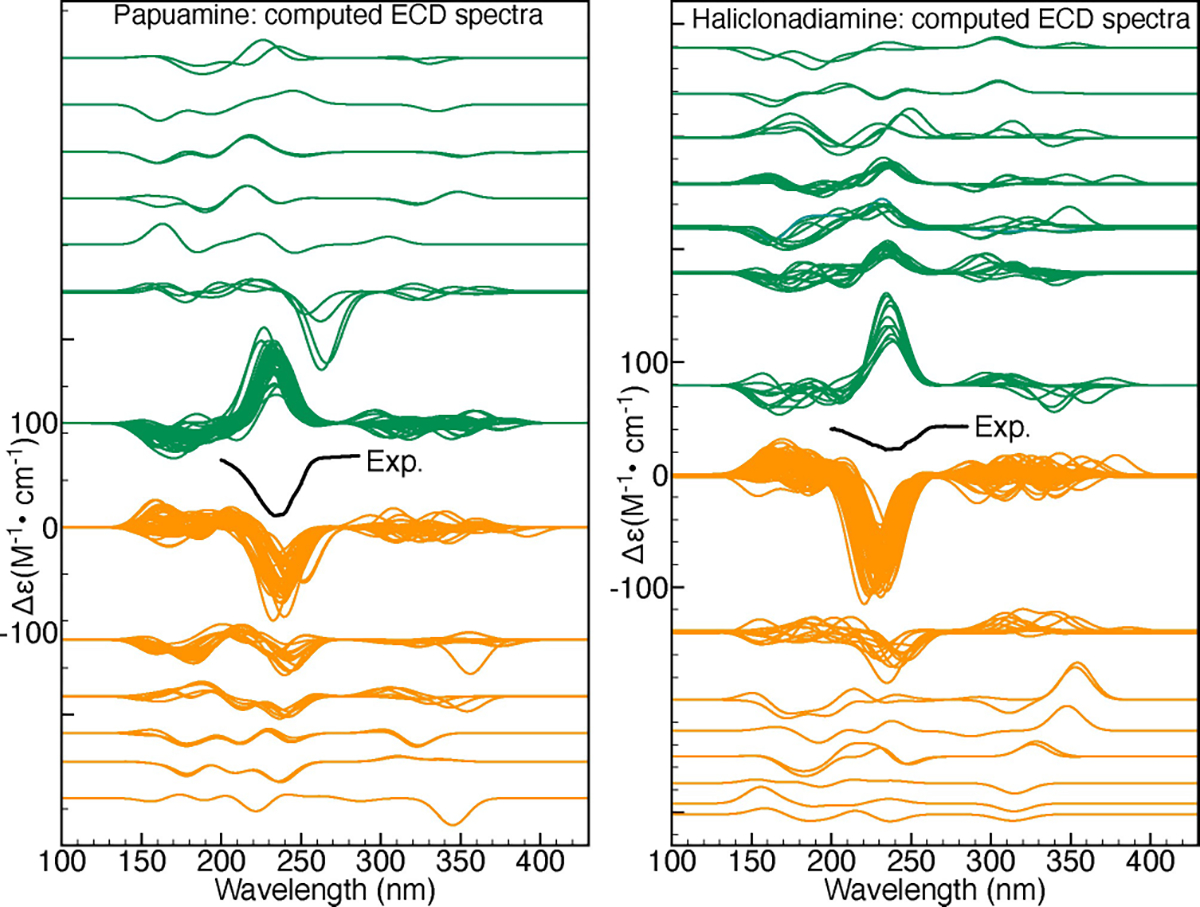
Families of ECD spectra organised according to the ECD dendrograms in Figures 7 and 8. The experimental ECD spectra measured in MeOH are also shown (in black).

To further clarify this point we compare the trends exhibited by the VCD and ECD spectra computed for the considered papuamine and haliclonadiamine conformers. Figure 7 shows the dendrograms associated with the similarities between the VCD and ECD spectra of the haliclonadiamine conformers, while Figure 8 shows the VCD and ECD dendrograms associated with the papuamine spectra. As can be seen in both Figures, there is a striking difference between the VCD and ECD dendrograms, with the VCD spectra presenting a significantly larger variation among the conformations that are considered. Put at a more quantitative level: taking a 0.4 pruning threshold, the VCD families are small and typically consist of 2–3 conformers, which are characterised by flipped cyclohexane groups (see Figure S9 in the SI). This demonstrates clearly the high sensitivity of VCD to small structure variations. ECD spectra, on the other hand, are determined by the conformation of the diene chromophore (see Figure S10 in the SI). As such, ECD families are typically large and consist of many conformers that have similar spectra even though their structures are different (see Figure 9).

If no pruning threshold is taken in the ECD dendrograms, the spectra of both molecules are grouped into only two ECD families. The connection between these two families occurs at 1.9 on the dendrogram y-axis. This corresponds to a TSI value of −0.9, which indicates that a significant number of spectra in one family have nearly mirror-image equivalents in the other as is also evident from Figure 9. This observation has far-reaching consequences. Since the experimental ECD spectra of haliclonadiamine and papuamine consist of a single negative band (around 230 nm) and the computed Boltzmann factors suffer from large uncertainties, one cannot use ECD spectroscopy to assign the absolute configuration of these two molecules in solutions by neither the standard nor the GA approach (see Figures S11 and S12 in the SI).

VCD does not suffer from this drawback. The experimental VCD spectra feature many bands in the fingerprint region. As a result, it is found that without pruning threshold the VCD spectra are grouped into 19 and 20 main families for haliclonadiamine and papuamine, respectively. Furthermore, the highest connection between the main VCD families is 1.29 for haliclonadiamine and 1.22 for the papuamine. Such values correspond to TSI values of −0.29 and −0.22, respectively, and imply that there are poor (negative) similarities between spectra. The experimental VCD spectra of haliclonadiamine and papuamine can therefore not be reproduced by calculations performed on the opposite enantiomer. This assures us that the AC assignments made with VCD is very reliable.

Lastly, we would like to assess to what extent the calculations are able to differentiate without prior knowledge the two diastereomers considered here. Consequently, we have simulated the experimental VCD spectrum of papuamine using the calculations for haliclonadiamine and vice versa. Table 5 shows that both experimental spectra can be reproduced by calculations performed for the other (incorrect) molecule, albeit that the computed spectra need to be multiplied by −1. Since 7 out of the 8 chiral centres have an opposite configuration in the two molecules and since the two structures technically differ only in the orientation of a single H atom, this is what one would indeed expect.

At the same time, we notice that the similarity indices computed when using the incorrect molecule are smaller than the ones obtained for the correct molecule. This shows that VCD is able to sense that there is a small difference between the structures of papuamine and haliclonadiamine. However, for practical purposes these differences are too small to facilitate an unambiguous discrimination between the two diastereomers and their enantiomers, or in other words, the differences between the GA-VCD spectra simulated using the correct and incorrect structures (see Figure S13 in the SI) are so small that one is unable to tell whether one is dealing with a diastereomer or with the other enantiomer.

## Conclusion

The primary limitation of the current protocol for determining the absolute configuration of chiral molecules using chiroptical spectroscopy is the uncertainty in the Boltzmann factors calculated using DFT. The studies performed here for papuamine and haliclonadiamine have revealed uncertainties of at least 5 kcal/mol for the computed relative energies. As a result, it was not possible to make a reliable assignment of the absolute configuration of these two compounds. By employing a combination of genetic and hierarchical clustering machine learning algorithms, we have successfully circumvented the shortcomings of the DFT Boltzmann factors enabling us to make highly reliable AC assignments. Unlike the standard protocol which relies blindly on the inaccurate DFT Boltzmann factors and often neglects important conformations, the approach proposed here performs a thorough analysis of the spectra of all considered conformers. This allows it (i) to solve difficult cases and (ii) to identify situations in which a particular chiroptical technique is intrinsically not able to provide a reliable prediction. Given the importance of correct AC determinations in chemical and biological sciences, this is of paramount importance. The approach presented here is quite general. It can therefore be used to analyse any type of molecular spectra and not just chiroptical spectra such as ECD, VCD, and ROA spectra. We thus expect that it will be highly rewarding to incorporate this approach in the analysis of spectra of complex systems in general.

## Artificial intelligence algorithms

**Figure 10.**
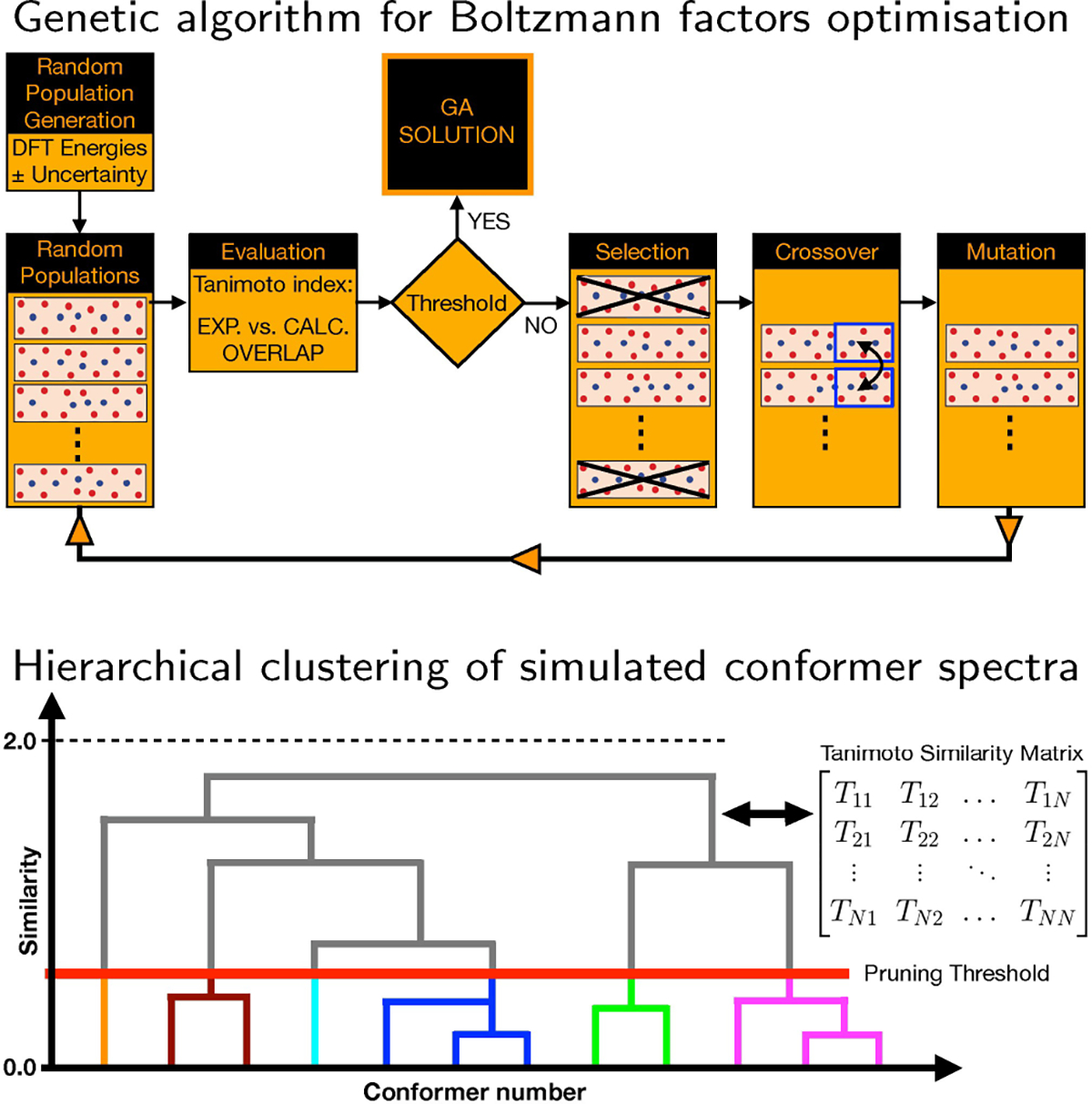
Schematic representation of the genetic and hierarchical clustering AI-algorithms employed for the analysis of chiroptical spectra.

In the following we will discuss the protocol that has been introduced to analyse chiroptical spectra. It is based on an in-house developed Python code that combines a genetic algorithm and a hierarchical clustering algorithm. Figure 10 shows schematically how these two algorithms are used.

A genetic algorithm is used to optimise the Boltzmann populations of the considered conformers such that an optimal agreement is obtained between the experimental and simulated spectra. Genetic algorithms are known to provide effective solutions for optimisation problems of this kind. They perform a heuristic type of search and require a fitness function to asses how good a given solution is. In the present studies the Tanimoto similarity index (referred to in the following as TSI), which is identical to the SymVCD index introduced by Shen et al.,^[16]^ was used as fitness function:

(1) $$T_{ij}=\frac{S(ij)}{S(ii)\;+\;S(jj)\;-\;|S(ij)|}$$

where $S\left(ij\right)$ is the overlap integral between the spectra i and j. The Tanimoto index ($T_{ij}$) is +1 when spectra i and j are identical and 1 when they are mirror images of each other. For this reason $T_{ij}$ is well suited to quantify how similar/different two chiroptical spectra are.

A schematic representation of the procedure employed for optimising the Boltzmann factors using a genetic algorithm is shown in the upper panel of Figure 10. Starting from the DFT energies, an ensemble of populations is generated by changing the energy of each conformer randomly with an amount that is smaller than a user-defined energy threshold. While each population in an ensemble contains the same number of members—namely, the number of considered conformers—the energies of the conformers differ randomly amongst the members of an ensemble. Based on these energies, Boltzmann-averaged spectra are computed and compared to the experimental spectrum. In the “Selection” step the populations whose spectra are sufficiently similar to the experimental spectrum are retained, while the other populations are discarded. In the “Crossover” step populations deemed as fit in the previous step are grouped into pairs (so-called parents) from which offspring are produced by randomly swapping some of the conformers between parents. Finally, in the “Mutation” step a small number of random mutations are induced in the offspring. In this way a new generation of populations is produced and the procedure is repeated till the similarity index between the experimental and simulated spectrum is not further improved.

An important aspect to consider is to what extent such fits can come to an unambiguous AC assignment. Clearly, this can only be done if there is a significant difference between Tanimoto indices obtained for the experimental spectrum and its mirror image. For this reason, it is necessary to verify that by running the genetic algorithm on a set of calculations performed for a given enantiomer, one cannot reproduce the experimental spectra of both enantiomers equally well, or, equivalently, that the experimental spectrum cannot be fitted equally well with the spectra calculated for the two enantiomers.

The genetic algorithm described above employs calculated spectra of selected conformers to reproduce the experimental spectrum. It is thus important to know how similar/different these spectra are, and in particular whether there are conformers or families of conformers whose spectra are almost mirror image of each other. To this purpose hierarchical clustering analyses have been performed. In such an analysis a similarity matrix is constructed by computing similarity Tanimoto indices between the simulated spectra of all considered conformers. The information contained in the similarity matrix is visualised as a dendrogram, i.e., a hierarchical tree diagram that groups the considered conformers into families (see lower panel of Figure 10). Conformers in a given family have similar spectra, while the conformers in different families have dissimilar spectra.

As shown in Figure 10 a dendrogram consists of different levels of stacked branches. The conformers in a given family (highlighted by colours in Figure 10) are connected by horizontal lines. The position of the horizontal lines with respect to the y-axis of the dendrogram indicates how similar the spectra of the connected conformers are. The y-axis takes values between 0, which indicates identical spectra (a Tanimoto index +1) and 2, which indicates mirror image spectra (a Tanimoto index −1). By defining a pruning threshold (see Figure 10), one can modify the number of families by defining how different the members of a family can be. Values of the pruning threshold of 0.4 or smaller, which correspond to a Tanimoto index of 0.6 or larger, yield families of conformers that have rather similar spectra.

The insight provided by such an analysis is key to interpret the results from the genetic algorithm fits. As shown in Figures 7, 8 and 9, it allows one to identify situations in which chiroptical spectroscopy techniques cannot make reliable AC assignments and also explains why this is the case.

## Supplementary Material

Supporting_Information

## Figures and Tables

**Table 1:** Dependence of the Tanimoto index on the energy uncertainty threshold (Δ*E*). The Tanimoto index was computed between the experimental (Exp.) VCD spectra of papuamine (P) and haliclonadiamine (H) and calculated (Calc.) spectra obtained using the genetic algorithm. VCD spectra were computed for 134 papuamine conformers and for 146 haliclonadiamine conformers. Frequency scaling factors used to simulate the experimental papuamine spectrum: 1.010 (950–1220 cm^−1^), 1.030 (1220–1400 cm^−1^) and 1.010 (1400–1500 cm^−1^). Frequency scaling factors used to simulate the experimental haliclonadiamine spectrum: 1.015 (950–1260 cm^−1^), 1.030 (1260–1400 cm^−1^) and 1.015 (1400–1500 cm^−1^). For Δ*E* = 0.0 the genetic algorithm computes the DFT Boltzmann weighted (DFT-BWed) spectrum. Δ*E* is given in kcal/mol. The “ −1 × P" and "−1 × H" simulated spectra were obtain by multiplying with −1 the rotational strengths computed for all papuamine and haliclonadiamine conformers.

Exp.:	P	H	P	H	
Calc.:	P	H	−1 × P	−1 × H	Δ*E*

	+0.167	+0.346	−0.167	−0.346	0.0
	+0.451	+0.503	−0.004	−0.133	1.0
	+0.488	+0.642	+0.082	−0.082	2.0
	+0.588	+0.712	+0.103	−0.080	3.0
	+0.659	+0.770	+0.104	+0.002	4.0
	+0.725	+0.780	+0.109	+0.018	5.0
	+0.736	+0.784	+0.116	+0.019	6.0
	+0.737	+0.785	+0.117	+0.019	7.0
	+0.737	+0.785	+0.104	+0.019	8.0
	+0.737	+0.785	+0.104	+0.019	9.0
	+0.737	+0.785	+0.114	+0.019	10.0

**Table 2:** Tanimoto similarity indices (TSI) and Boltzmann weights (BW) of the most relevant haliclonadiamine conformers: predictions made by DFT (upper section) vs. predictions made by the GA-VCD protocol (lower section). All relative energies (Δ*E*) are computed with respect to conformer 2 (i.e., the lowest energy according to DFT) using the DFT free Gibbs energies. Relative energies are given in kcal/mol. The Tanimoto indices give the similarity between the experimental VCD spectrum and the DFT VCD spectra of the individual conformers. The 5th column indicates whether or not a given conformer exhibits a N-H...N-H intramolecular hydrogen bond.

Conf.	DFT-BWs	TSI	Δ*E*	H-bond

2	0.37	0.24	0.00	yes
26	0.23	0.19	0.28	yes
12	0.16	0.15	0.48	yes
25	0.11	0.09	0.70	yes
28	0.05	0.15	1.15	yes
24	0.04	0.25	1.31	yes

Total:	0.96			

Conf.	GA-BWs	TSI	Δ*E*	H-bond

40	0.19	0.52	6.60	no
151	0.12	0.31	5.78	no
55	0.09	0.39	4.58	no
81	0.08	0.29	4.50	yes
1	0.07	0.37	5.97	yes
6	0.06	0.27	3.87	yes
64	0.06	0.26	2.44	no
121	0.06	0.31	4.78	no
180	0.06	0.27	7.95	yes
199	0.06	0.23	5.56	yes
137	0.03	0.33	7.58	no
17	0.02	0.22	9.28	no
24	0.02	0.25	1.31	yes
227	0.02	0.12	9.32	no
19	0.01	0.12	9.58	no
2*	0.01	0.24	0.00	yes
66*	0.01	0.25	4.10	no
110*	0.01	0.21	9.17	yes
148*	0.01	0.24	5.95	no
186*	0.01	0.14	9.56	no

Total:	1.00			

**Table 3:** Prediction made by various levels of theory for the relative energy (*E*) of haliclonadiamine conformers 2 and 40. The relative energies are given in kcal/mol and were computed using the free Gibs energies.

Theory level	*E*_40_–*E*_2_

DFT, Vacuum, BP86, DZP	6.29
DFT, Vacuum, BP86, TZP	6.60
DFT, Vacuum, BP86, TZ2P	6.60

DFT, Vacuum, B3LYP, DZP	5.98
DFT, Vacuum, B3LYP, TZP	6.15
DFT, Vacuum, B3LYP, TZ2P	6.15

DFT, COSMO, BP86, DZP	4.55
DFT, COSMO, BP86, TZP	4.61
DFT, COSMO, BP86, TZ2P	4.67

DFT, COSMO, B3LYP, DZP	4.19
DFT, COSMO, B3LYP, TZP	4.14
DFT, COSMO, B3LYP, TZ2P	4.22

DFTD, Vacuum, BP86, DZP	5.38
DFTD, Vacuum, BP86, TZP	5.79
DFTD, Vacuum, BP86, TZ2P	5.80

DFTD, COSMO, BP86, DZP	3.55
DFTD, COSMO, BP86, TZP	3.67
DFTD, COSMO, BP86, TZ2P	3.76

**Table 4:** Dependence of the GA-VCD Boltzmann weights (GA-BWs) on the signal-to-noise ratio of the experimental VCD spectrum. Two different experimental VCD spectra recorded for 24 and 6 hours, respectively, were used when running the genetic algorithm. TSI values were computed for the 1050–1500 cm^−1^ interval.

Conf.	GA-BWs (24 h)	GA-BWs (6 h)

40	0.19	0.07
55	0.08	0.17
151	0.08	0.10
180	0.07	0.03
137	0.06	0.10
199	0.04	0.04
81	0.04	0.07
24	0.02	0.09
60	0.01	0.11
2	0.01	0.01
6	0.01	0.01
14	0.01	0.03

1	0.08	-
121	0.06	-
148	0.04	-
64	0.03	-
110	0.03	-
227	0.02	-
17	0.02	-
116	0.02	-
133	0.01	-
202	0.01	-
216	0.01	-
33	-	0.06
109	-	0.06
75	-	0.02
12	-	0.01
26	-	0.01

TSI	0.82	0.63

**Table 5:** Diastereomer discrimination using the GA protocol. An energy uncertainty of 5 kcal/mol was used in all simulations. Frequency scaling factors required to reproduce the experimental spectrum of Papuamine (P): 1.010 (1050–1220 cm^−1^), 1.030 (1220–1400 cm^−1^), 1.010 (1400–1550 cm^−1^). Frequency scaling factors required to reproduce the experimental spectrum of Haliclonadiamine: (H) 1.015 (950–1260 cm^–1^), 1.030 (1260–1400 cm^–1^), 1.015 (1400–1500 cm^−1^). The “ −1 × P” and "1 × H” indicate that the rotational strengths of the respective calculations have been multiplied by minus one.

Exp.	Calc.	TSI

P	P	+0.7252
P	−1 × H	+0.5483
H	H	+0.7802
H	−1 × P	+0.6958

## Data Availability

The data that support the findings of this study are available from the corresponding author upon reasonable request.
