# Supplementary material for: An Artificial Intelligence Approach for Tackling Conformational Energy Uncertainties in Chiroptical Spectroscopies
Source: Angew Chem Int Ed Engl. Author manuscript; Available in PMC 2024 Sep 22. (PMC11416722; doi:10.1002/anie.202307053)
Supplement: Supporting_Information [file NIHMS2020879-supplement-Supporting_Information.pdf]

Angewandte Chemie
Eine Zeitschrift der Gesellschaft Deutscher Chemiker

Supporting Information

An Artificial Intelligence Approach for Tackling Conformational Energy Uncertainties in Chiroptical Spectroscopies

G. Marton, M. A. J. Koenis, H.-B. Liu, C. A. Bewley, W. J. Buma, V. P. Nicu**

1 Experimental details

To obtain the free base form of papuamine and haliclonadiamine for VCD measurements, an ca. 1 g sample of the organic extract of *Haliclona* sp. (sample number C29959 from the NCI Open Repository) shown previously to contain both natural products[1] was suspended in MeOH (5 mL) and stirred at room temperature for 30 min. The cloudy solution was centrifuged at 6,000 rpm for 1 hr and the supernatant was injected in ca. 5 mg aliquots onto a Phenomenex Lux 5 μm Cellulose-1 column (10 x 250 mm) equilibrated in 1:1 MeOH:2-propanol with 0.1% diethylamine, and eluting using the same isocratic conditions. Fractions containing papuamine or haloclonadiamine were combined and the solvent removed in vacuo. Samples were relyopholized two additional times after resuspending in MeOH:H_2O and flash freezing in liquid N_2. The separations yielded approximately 15 mg of the free base of each compound. Therefore, unlike the previous investigation[1], which has studied the doubly protonated compounds, here we are considering the neutral compounds.

Vibrational absorption (VA) and VCD spectra were recorded with a Biotools Chiral-2X spectrometer. The samples were measured for 24 hours using a 205 μm BaF_2 sample cell. Papuamine was measured in CD_2Cl_2 at a concentration of 0.134 M, while haliclonadiamine was measured in $CDCl_3$ at a concentration of 0.125 M. The spectrum of the pure solvents was used as a baseline for the VA and VCD spectra.

2 Computational details

Conformational searches were performed using the RDkit software package[2]. For each molecule, ten thousand conformations were generated and optimised using the universal forcefield (UFF)[3], then filtered for duplicates using an RMS-threshold of 0.1 Å. Subsequently, UFF conformers within an energetic window of 15 kcal/mol (200 and 230 for papuamine and haliclonadiamine, respectively) were optimised with Density Functional Theory (DFT)[4] using a very stringent convergence threshold, (10^{-5} Hartree/Angstrom) for the nuclear gradients. Since many of the UFF conformers have converged to the same minimum, a total of 134 unique papuamine conformers and 146 unique haliclonadiamine conformers were found after the DFT geometry optimisations. The set of unique conformers of each molecules was found to span an energy interval of approximately 10 kcal/mol. VCD and ECD spectra were computed using DFT for all the distinctive papuamine and haliclonadiamine conformers. The VCD[5] and ECD calculations were performed using the Amsterdam Density Functional (developer version r78297, 2019-10-07) software package[6–8] using the TZP/BP86 level of theory[9–11]. ECD spectra were computed also with the CAM-B3LYP[12] exchange-correlation functional, but only for the conformations with room temperature populations that were larger than 1%.

To test the uncertainty of the computed relative energies, the UFF conformers of hal-

iclonadiamine were relaxed also using the COSMO continuum and the DFTD models (which accounts for London dispersion interactions) in combination with the BP86 and B3LYP[13,14] functionals and three different basis sets DZP, TZP and TZ2P.

The computed VA and VCD intensities were convoluted with a Lorentzian function using a half width at half maximum of 4 cm^{-1}. To obtain optimal overlaps between the simulated and experimental spectra, two different scaling factors were used for the normal mode frequencies. Moreover, because the papuamine and haliclonadiamine measurements were done in different solvents, slightly different factors were needed to simulate the two experimental spectra. For papuamine scaling factors of 1.030 for the modes in the 1220–1400 cm^{-1} region and 1.010 for the modes in the 1050–1220 and 1400–1500 cm^{-1} regions were employed. For haliclonadiamine, on the other hand, 1.030 was used for the 1260–1400 cm^{-1} region and 1.015 for the 950–1260 and 1400–1500 cm^{-1} regions. These scaling factors were determined based on the analysis described in Section 2 below.

3 Dependence of the GA Boltzmann factors on frequency scaling factors and fitness functions

The results obtained when optimizing the Boltzmann factors using the genetic algorithm depend sensitively on the factors used to scale the frequencies of the normal modes and on the fitness function used by the genetic algorithm. To investigate this further we consider in this section four different scaling factors (labeled as A, B, C and D) and three different fitness functions (TSI(IR), TSI(VCD) and TSI(VCD)×TSI(IR)). When TSI(IR) is used as fitness function, the experimental IR spectrum is considered as the reference spectrum and the genetic algorithm optimizes the Boltzmann factors so that the highest value is obtained for the Tanimoto similarity index between the experimental and simulated IR spectra. When TSI(VCD) is used, the experimental VCD spectrum is used as reference and the goal of the genetic algorithm is to maximise the similarity between experimental and simulated VCD spectra. Finally, when the TSI(VCD)×TSI(IR) is used as fitness function, the goal of the genetic algorithm is to optimise the similarity between the IR and VCD spectra simultaneously.

The four sets of frequency scaling factors considered in this section are defined in Table S-I. Set A was chosen such that the largest TSI value is obtained between the experimental and simulated VCD spectra with the precise values of these scaling factors determined manually by trial and error runs of the genetic algorithms. From previous studies it is known that these scaling factors often work well when using the BP86/TZP level of theory. The B scaling factors were chosen to yield the largest cross-correlation TSI value, i.e., TSI(VCD)×TSI(IR), between the experimental and simulated spectra. The C scaling factors were chosen to yield the largest TSI value between the experimental and simulated IR spectra, while the D scaling factor is the linear scaling factor that yielded the largest

	Scaling factors			
Freq. interval (cm^{-1})	A	B	C	D
800.0 – 1260.0	1.015	1.015	1.025	1.015
1260.0 – 1400.0	1.030	1.030	1.030	1.015
1400.0 – 1900.0	1.015	1.005	1.005	1.015

Table S-I: Factors used to scale the frequencies of the normal modes. The A scaling factor yields the largest TSI value between the experimental and simulated VCD spectra; the B scaling factor the largest cross correlation TSI value, i.e., TSI(VCD)×TSI(IR) between the experimental and simulated spectra; the C scaling factors the largest TSI value between the experimental and simulated IR spectra; while The D scaling factor is the linear scaling factor that yields the largest TSI value between the experimental and simulated VCD spectra.

TSI value between the experimental and simulated VCD spectra.

Figure S1 shows a comparison of the GA Boltzmann factors obtained when using the three fitness functions in combination with the four frequency scaling factors, while Table S-II lists the associated TSI values. In the following we will refer to the combination between a given fitness function and the frequency scaling factors chosen to maximize the TSI value as a reference combination. In Figure S1 and Table S-II the numbers associated with the four reference combinations are highlighted by colored fonts. Tables S-III, S-IV and S-V list comparisons between the GA Boltzmann factors obtained for the four reference combinations, while Table S-VI lists the conformers that were identified having a room-temperature population of at least 1% by all reference combinations. Figure S2 shows comparisons of the experimental IR and VCD spectra with the simulated Boltzmann averaged spectra computed using the GA populations predicted by the four reference combinations.

These series of analyses lead to a few important observations. Focusing in first instance on the results obtained when using scaling factor C chosen to maximise TSI(IR), we find that while this choice yields that largest TSI(IR) value, it also yields the smallest TSI(VCD) values. This shows that for this particular molecule choosing the "common-sense" scaling choice frequency scaling factors that yield the best alignment between the experimental and simulated IR bands is not beneficial for VCD. This happens because the different intensity distribution over coalescing bands in the IR and VCD spectra result in a non-perfect alignment of the spectra. In addition, it should be noticed that since IR spectra are significantly less sensitive to the molecular conformation than VCD spectra, the use of TSI(IR) as the fitness function for the genetic algorithm yields the poorest conformer resolution. As can be seen in panel C in Figure S1 and Tables S-IV and S-VI, this method yields consistently the smallest number of relevant conformers and as a result the smallest

Scaling factor	Fitness functions						
	TSI(IR)	TSI(VCD)	TSI(VCD)×TSI(IR)	=	TSI(VCD)	×	TSI(IR)
A	0.7209	0.7802	0.5050	=	0.7631	×	0.6617
B	0.9020	0.7714	0.6400	=	0.7656	×	0.8360
C	0.9173	0.5738	0.4729	=	0.5663	×	0.8351
D	0.7034	0.7513	0.4687	=	0.6456	×	0.7259

Table S-II: Dependence of the Tanimoto similarity index (TSI) computed between experimental and simulated spectra on: (1) the factors used to scale the frequencies of the normal modes, and (2) the fitness function used by the genetic algorithm. Four different scaling factors (A, B, C and D; see Table S-I) were combined with three different fitness functions, TSI(IR), TSI(VCD) and TSI(VCD)×TSI(IR). The reference combinations have been highlighted by coloured fonts.

similarity between experimental and simulated VCD spectra.

Next, we discuss the differences between the results obtained with the TSI(VCD) and TSI(VCD)×TSI(IR) fitness functions. When using the A and B scaling factors, which differ only in the 1400-1500 cm^{-1} interval, these two fitness functions give rise to rather similar results as can be seen in Table S-II where very similar TSI(VCD) values are found for the two methods. Moreover, the comparison in Table S-III shows that the TSI(VCD)/A and TSI(VCD)×TSI(IR)/B reference combinations predict 20 and 21 relevant conformers, respectively. From these conformers, 14 were identified by both methods with the populations of these common conformers adding up to 91% and 81%, respectively. When the linear scaling D factor is employed, the TSI(VCD) fitness functions still exhibit a high level of similarity between the VCD spectra, but there is a decrease in similarity when TSI(VCD)×TSI(IR) is used. As can be seen in Table S-V, the two fitness functions still predict 10 common conformers but their populations are more distinct and their combined totals are only 59% and 63%, respectively.

To summarise the result of this analysis, we compiled in Table S-VI a list with the conformers that were identified as relevant when using the TSI(VCD)/A , TSI(VCD)×TSI(IR)/B and TSI(VCD)/D reference combinations. It is noteworthy that 7 conformers were identified as relevant by all three methods, with a combined population of 44%, 45%, and 43%. Additionally, we note that 4 of these 7 conformers were also identified as relevant with the TSI(IR)/C reference combination.

We therefore conclude that the predictions made by the GA protocol for the relevant conformers of haliclonadiamine are consistent and robust across a wide range of key parameters. Finally, we notice that in the main manuscript we have chosen the TSI(VCD) fitness function in combination with the scaling factor A as the reference method.The reason for this is threefold: (1) this combination yields the largest TSI values for the

VCD spectra, (2) the set A of scaling factors often works well when using the BP86/TZP level of theory and consists of only two factors unlike scaling set D which contains 3 factors, and (3) the interpretation of the results predicted by the GA approach is more straightforward when using a simpler fitness function.

4 Basis set dependence of structures and spectra

The structures and spectra predicted for the seven relevant conformers of haliclonadiamine (see Table S-VI) using the BP86 exchange-correlation functional and the DZP, TZP and TZ2P basis sets are compared in Figures S3–S5. As expected [5], the three sets of basis functions yield structures and spectra of very similar quality. The reason for this is twofold. Firstly, the adoption of a rigorous convergence threshold in the geometry optimization calculations has compelled all three sets of calculations to converge to the same minima on the potential energy surface. Secondly, this outcome should also be attributed to the fact that our basis functions are based on Slater type orbitals, which are renowned for producing consistent results with rapid convergence.

References

[1] H.-B. Liu, G. H. Imler, K. K. Baldridge, R. D. O'Connor, J. S. Siegel, J. R. Deschamps and C. A. Bewley, *J. Am. Chem. Soc.*, 2020, **142**, 2755 – 2759.

[2] *RDKIT: Open-source cheminformatics*, http://www.rdkit.org (accessed March 6, 2017).

[3] A. K. Rappé, C. J. Casewit, K. Colwell, W. Goddard III and W. Skiff, *J. Am. Chem. Soc.*, 1992, **114**, 10024–10035.

[4] M. Swart and F. M. Bickelhaupt, *J. Comput. Chem*, 2007, **29**, 724 – 734.

[5] V. P. Nicu, J. Neugebauer, S. K. Wolff and E. J. Baerends, *Theor. Chem. Acc.*, 2008, **119 (1-3)**, 245 – 263.

[6] *Amsterdam Density Functional program*, Theoretical Chemistry, Vrije Universiteit, Amsterdam, URL: http://www.scm.com.

[7] G. T. Velde, F. M. Bickelhaupt, E. J. Baerends, C. F. Guerra, S. J. A. V. Gisbergen, J. G. Snijders and T. Ziegler, *J. Comput. Chem.*, 2001, **22**, 931 – 967.

[8] C. F. Guerra, J. Snijders, G. te Velde and E. Baerends, *Theor. Chem. Acc.*, 1998, **99**, 391 – 403.

[9] E. van Lenthe and E. Baerends, *J. Comput. Chem.*, 2003, **24**, 1142 – 1156.

[10] A. D. Becke, *Phys. Rev. A*, 1988, **38**, 3098 – 3100.

[11] J. P. Perdew, *Phys. rev. B*, 1986, **33**, 8822 – 8824.

[12] T. Yanai, D. P. Tew and N. C. Handy, *Chem. Phys. Lett.*, 2004, **393**, 51–57.

[13] A. D. Becke, *J. Chem. Phys.*, 1983, **98**, 5648 – 5652.

[14] P. J. Stephens, F. J. Devlin, C. F. Chabalowski and M. J. Frisch, *J. Phys. Chem.*, 1994, **98**, 11623 – 11627.

		GA-BWs		
Conf.	$\Delta E(DFT)$	TSI(VCD)/A	TSI(VCD)×TSI(IR)/B	H-bonding
40	6.60	0.19	0.16	no
151	5.78	0.12	0.07	no
55	4.58	0.09	0.09	no
81	4.50	0.08	0.08	yes
1	5.97	0.07	0.06	no
6	3.87	0.06	0.04	no
64	2.44	0.06	0.02	no
121	4.78	0.06	0.04	no
180	7.95	0.06	0.06	yes
199	5.56	0.06	0.06	yes
17	9.28	0.02	0.03	no
227	9.32	0.02	0.03	no
2	0.00	0.01	0.01	yes
66	4.10	0.01	0.06	no
137	7.58	0.03	–	no
24	1.31	0.02	–	yes
19	9.58	0.01	–	no
110	9.17	0.01	–	yes
148	5.95	0.01	–	no
186	9.56	0.01	–	no
60	8.69	–	0.08	no
72	6.82	–	0.05	no
205	8.95	–	0.03	no
26	0.28	–	0.01	yes
57	5.89	–	0.01	yes
191	7.11	–	0.01	no
202	5.51	–	0.01	no

Table S-III: Comparison of the GA-VCD Boltzmann weights (GA-BWs) obtained using set A of frequency scaling factors in combination with the TSI(VCD) fitness function and those obtained using set B in combination with the TSI(VCD)×TSI(IR) fitness function.

		GA-BWs		
Conf.	$\Delta E(DFT)$	TSI(VCD)/A	TSI(IR)/C	H-bonding
199	5.56	0.06	0.43	yes
121	4.78	0.06	0.16	no
17	9.28	0.02	0.02	no
66	4.10	0.01	0.06	no
2	0.00	0.01	0.01	yes
1	5.97	0.07	–	no
6	3.87	0.06	–	no
19	9.58	0.01	–	no
24	1.31	0.02	–	yes
40	6.60	0.19	–	no
55	4.58	0.09	–	no
64	2.44	0.06	–	no
81	4.50	0.08	–	yes
110	9.17	0.01	–	yes
137	7.58	0.03	–	no
148	5.95	0.01	–	no
151	5.78	0.12	–	no
180	7.95	0.06	–	yes
186	9.56	0.01	–	no
227	9.32	0.02	–	no
48	7.86	–	0.19	no
53	3.97	–	0.04	no
60	8.69	–	0.05	no
92	9.88	–	0.01	no
180	7.95	–	0.01	yes
191	7.11	–	0.02	no

Table S-IV: Comparison of the GA-VCD Boltzmann weights (GA-BWs) obtained using set A of frequency scaling factors in combination with the TSI(VCD) fitness function and those obtain using set C in combination with the TSI(IR) fitness function.

Conf.	$\Delta E(DFT)$	GA-BWs TSI(VCD)/A	GA-BWs TSI(VCD)/D	H-bonding
40	6.60	0.19	0.04	no
151	5.78	0.12	0.09	no
55	4.58	0.09	0.05	no
199	5.56	0.06	0.09	yes
121	4.78	0.06	0.08	no
24	1.31	0.02	0.03	yes
227	9.32	0.02	0.02	no
2	0.00	0.01	0.01	yes
66	4.10	0.01	0.14	no
148	5.95	0.01	0.08	no
81	4.50	0.08	–	yes
1	5.97	0.07	–	no
6	3.87	0.06	–	no
64	2.44	0.06	–	no
180	7.95	0.06	–	yes
137	7.58	0.03	–	no
17	9.28	0.02	–	no
19	4.10	0.01	–	no
110	9.17	0.01	–	yes
186	9.56	0.01	–	no
36	7.91	–	0.07	yes
42	5.47	–	0.07	no
67	7.49	–	0.06	no
144	8.67	–	0.06	no
5	8.17	–	0.02	no
48	7.86	–	0.02	no
76	7.54	–	0.02	no
18	7.11	–	0.01	no
60	8.69	–	0.01	no
150	7.49	–	0.01	no
193	7.61	–	0.01	no

Table S-V: Comparison of the GA-VCD Boltzmann weights (GA-BWs) obtained using set A of frequency scaling factors in combination with the TSI(VCD) fitness function and those obtain using set D in combination with the TSI(VCD) fitness function.

	GA-BWs				
Conf.	Scaling A	Scaling B	Scaling D	Scaling C	H-bonding
40	0.19	0.16	0.04	–	no
55	0.09	0.09	0.05	–	no
199	0.06	0.06	0.09	0.43	yes
121	0.06	0.04	0.08	0.16	no
227	0.02	0.03	0.02	–	no
2	0.01	0.01	0.01	0.01	yes
66	0.01	0.06	0.14	0.06	no
Sum	0.44	0.45	0.43	0.66	–

Table S-VI: Dependence of the GA Boltzmann population on the frequency scaling factors of normal modes. Conformers are reported that have a room-temperature population of at least 1% when using sets A, B and D. Four of these conformers have been found to be relevant as well when using set C. The sets A, B, C and D of scaling factors are defined in Table S-I.

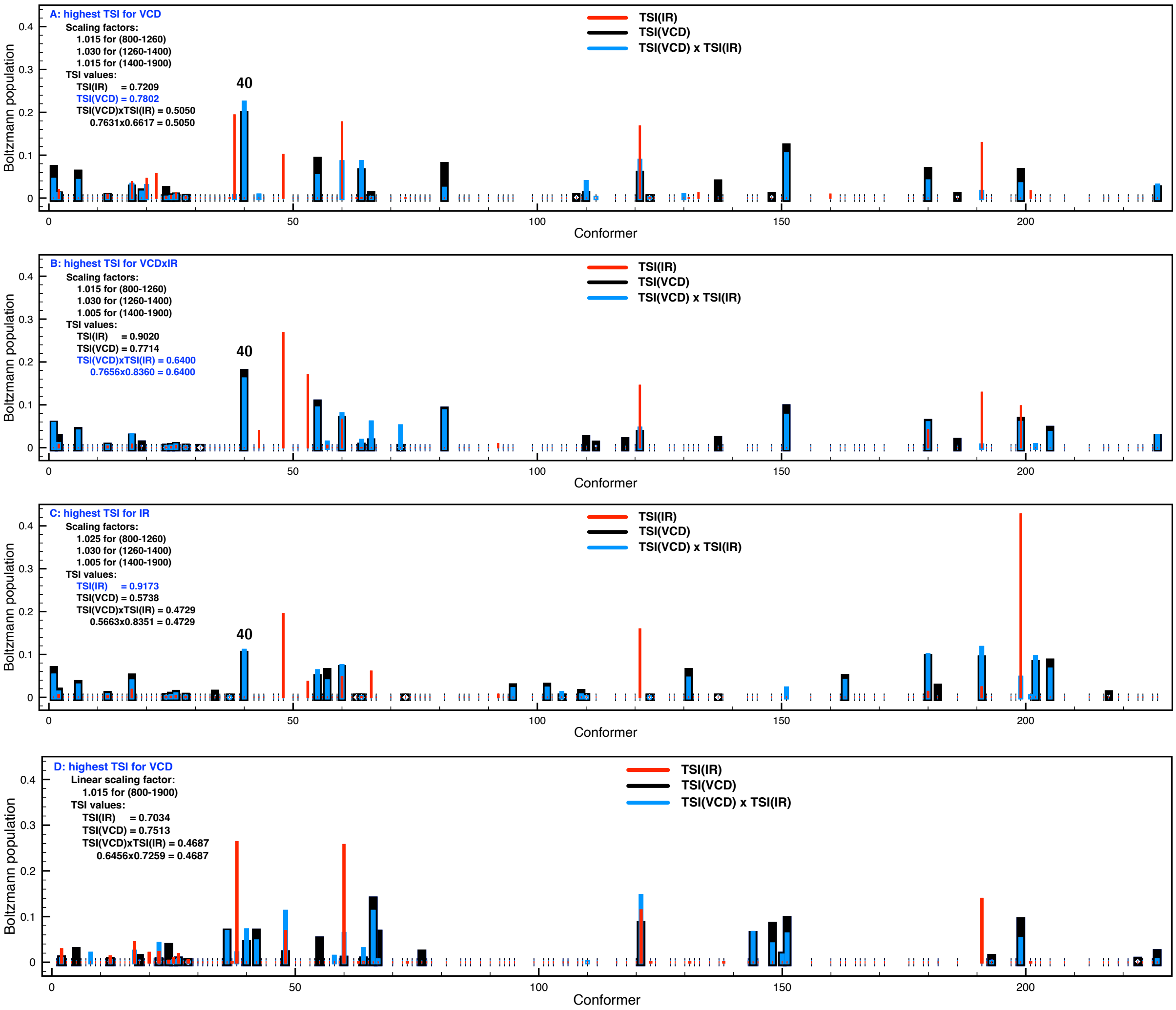

Figure S1: Dependence of the GA Boltzmann factors on: (1) the fitness function used during the GA optimisation, and (2) the factors used for scaling the frequencies of the normal modes. Three different fitness functions have been used: TSI(IR), TSI(VCD) and TSI(VCD)×TSI(IR). When using the TSI(IR) as fitness function, the genetic algorithm optimises the Boltzmann factors such that the largest Tanimoto similarity index (TSI) is obtained between the experimental and simulated IR spectra. When TSI(VCD) is used, the Boltzmann factors are optimised to increase the similarity between the experimental and simulated VCD spectra, while when TSI(VCD)×TSI(IR) is used the goal is increase the similarity between the IR and VCD spectra simultaneously. Four different scaling factors (A, B, C and D) were used, they are listed in the in this figure and also defined in Table S-I.

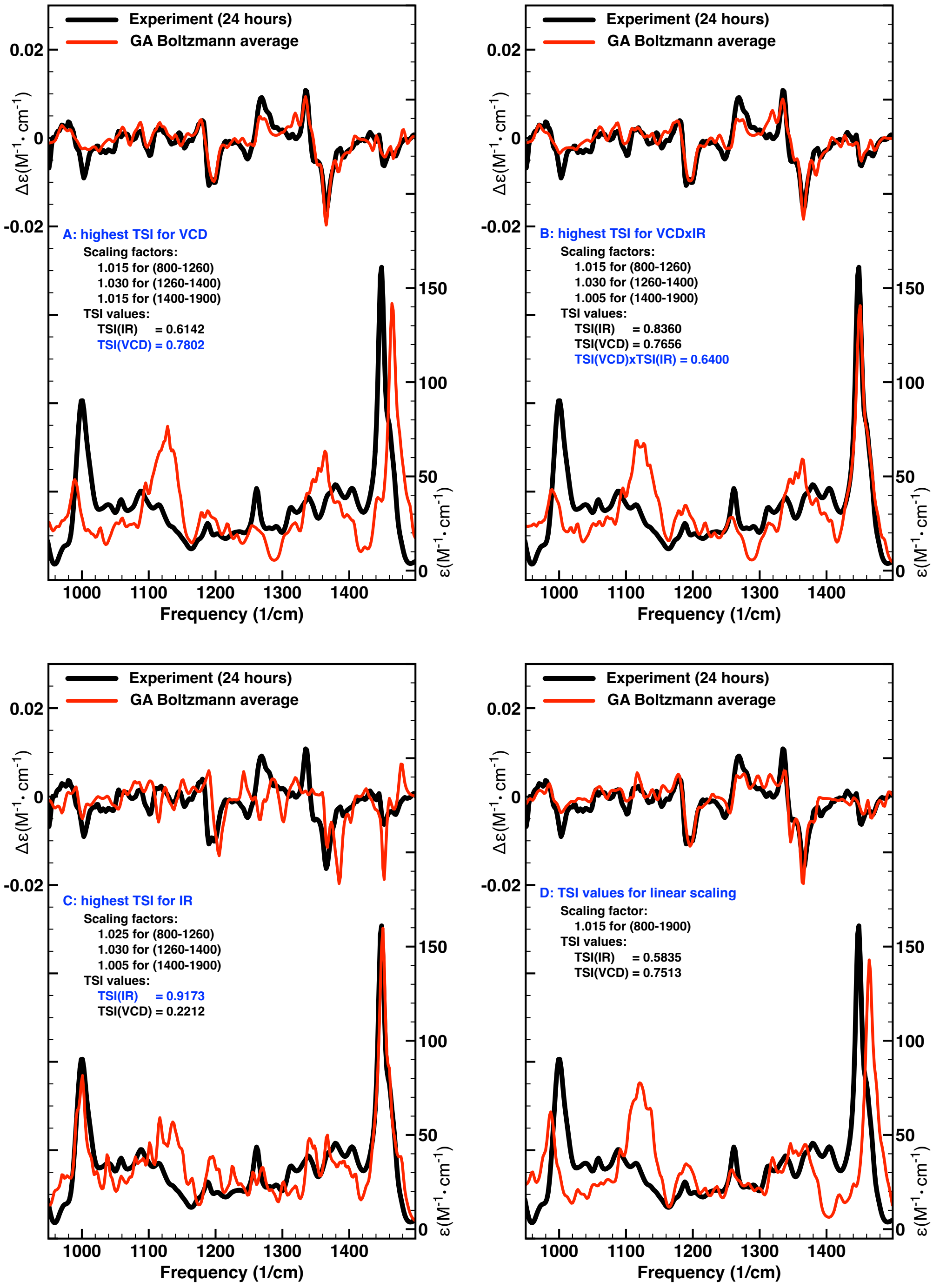

Figure S2: Comparison of experimental and simulated IR and VCD spectra. The simulated spectra are Boltzmann averaged spectra obtained using the GA populations predicted by the four reference combinations of fitness functions and frequency scaling factors: TSI(VCD)/A, TSI(VCD)×TSI(IR)/B, TSI(IR)/C and TSI(VCD)/D.

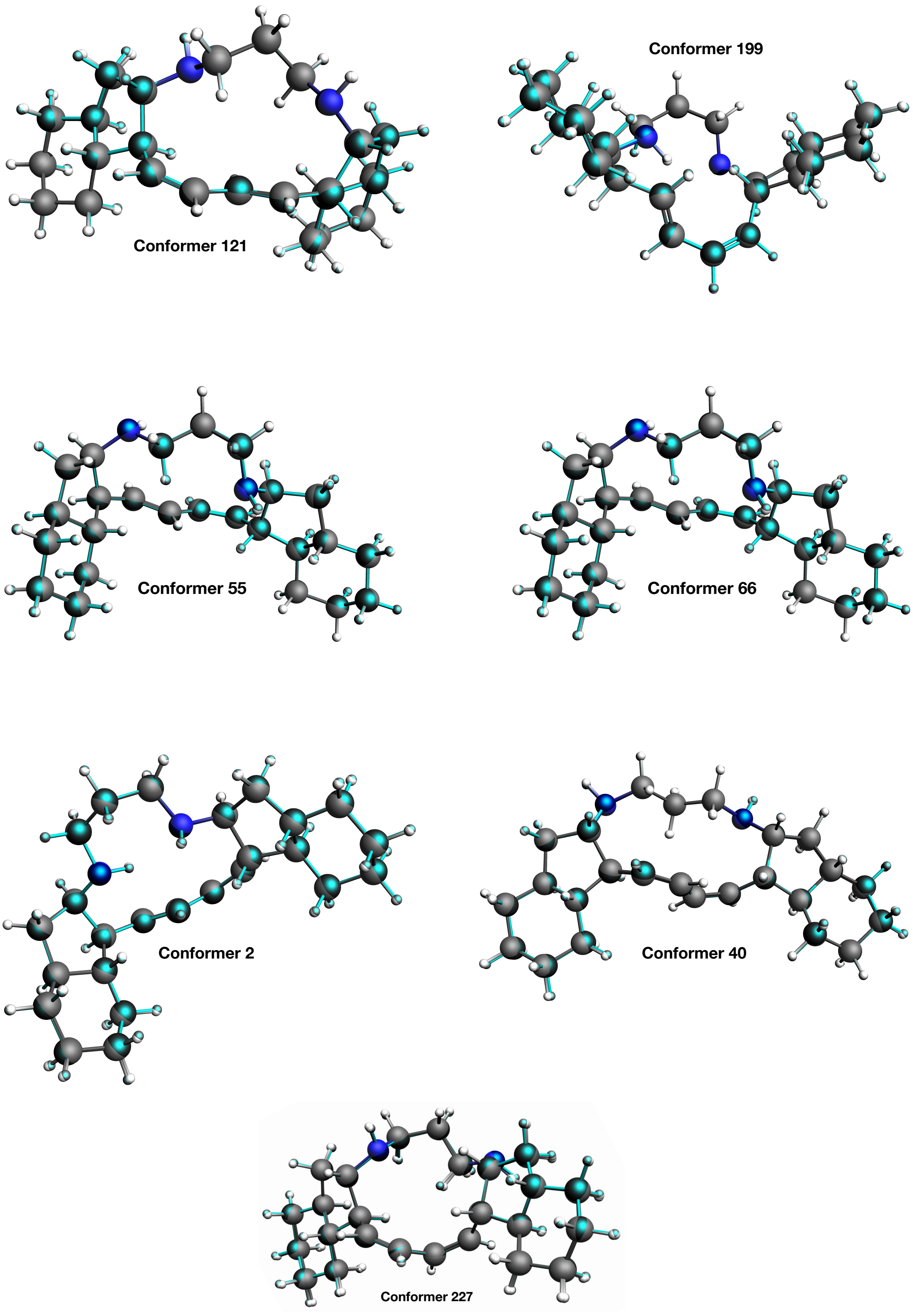

Figure S3: Superimposed DZP, TZP and TZ2P structures of the relevant low-energy conformers listed in Table S-VI. The TZP structures are highlighted in green. Minor differences can be observed for the H atoms, which often exhibit slightly different orientations.

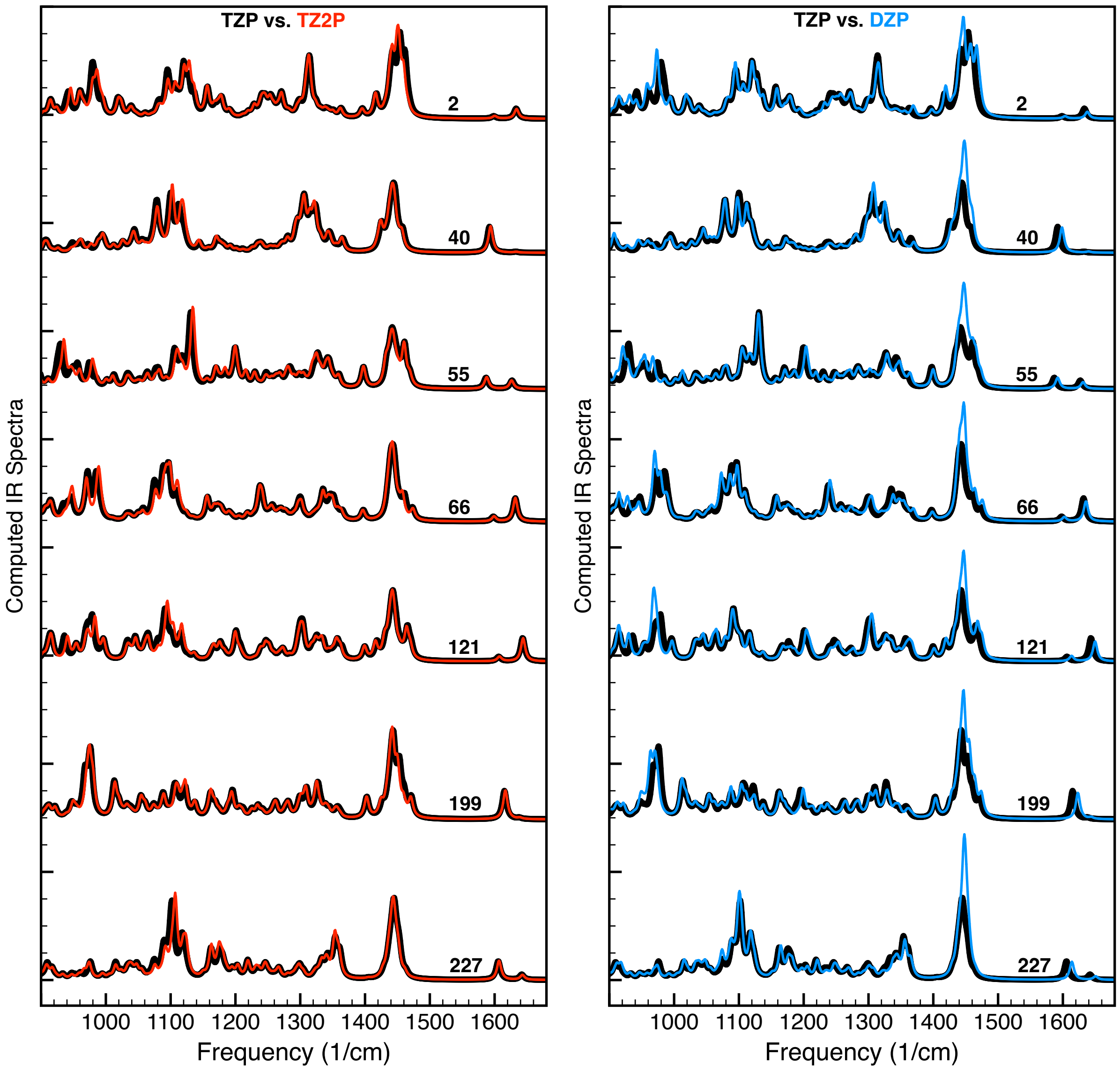

Figure S4: Comparison of the IR spectra computed using the DZP, TZP and TZ2P basis sets for the relevant low-energy conformers listed in Table S-VI.

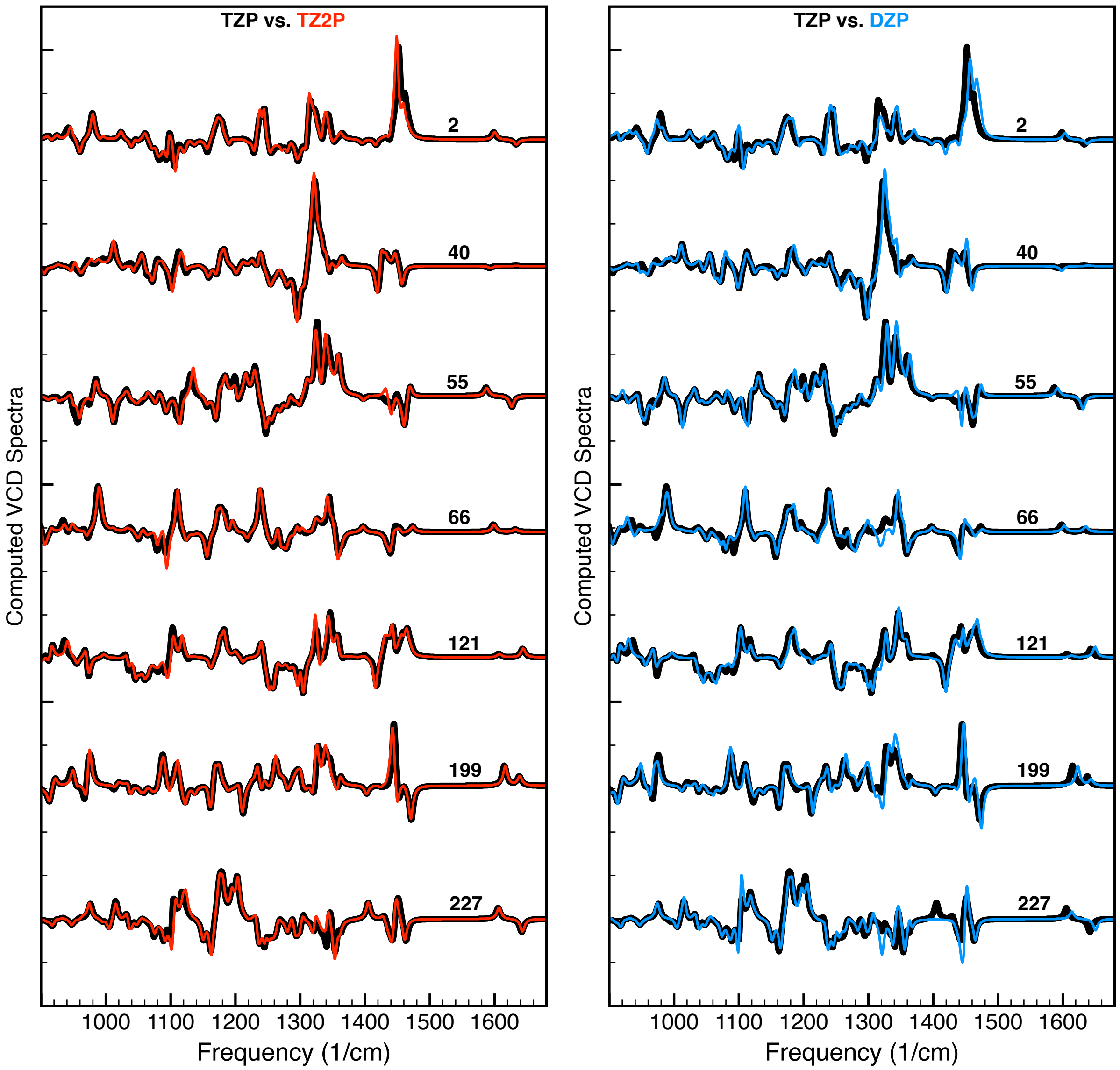

Figure S5: Comparison of the VCD spectra computed using the DZP, TZP and TZ2P basis sets for the relevant low-energy conformers listed in Table S-VI.

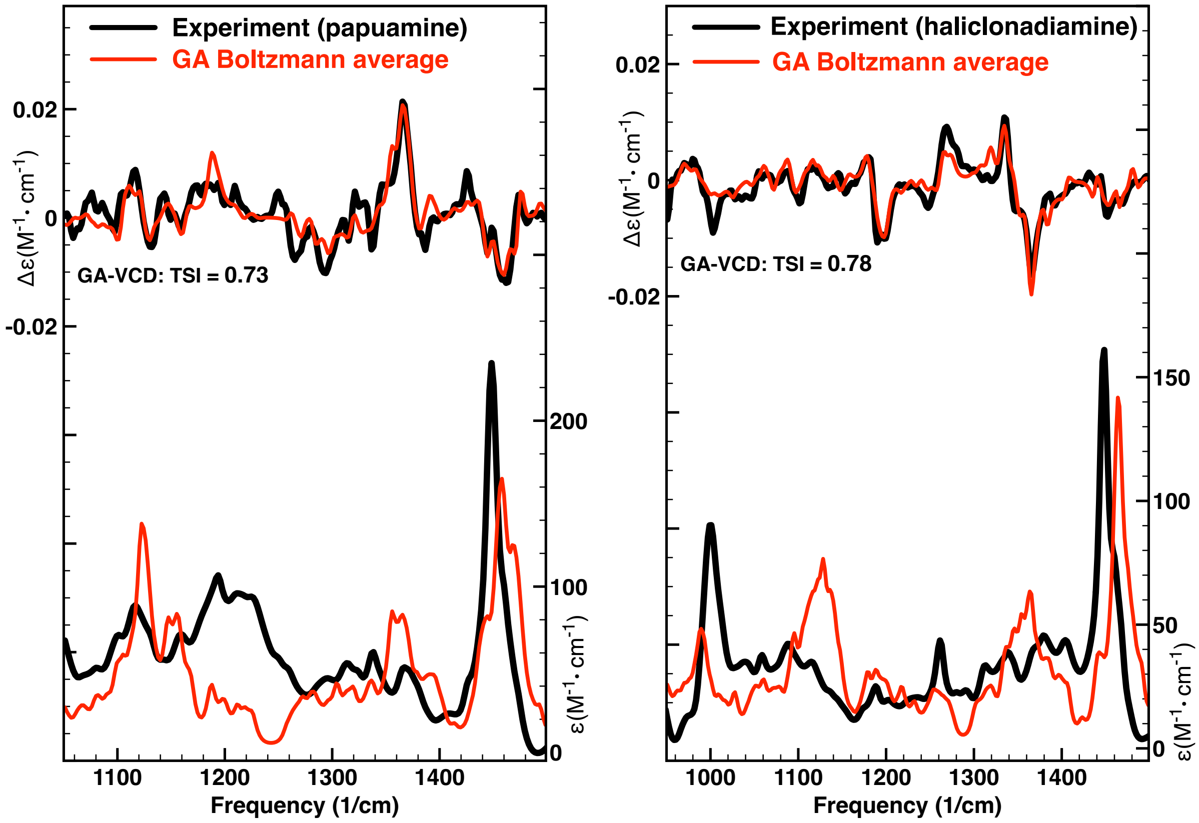

Figure S6: Comparison of experimental and simulated IR and VCD spectra of papuamine and haliclonadiamine. The simulated spectra have been obtained by averaging the spectra computed for the considered conformers using the Boltzmann factors optimised using the GA-VCD protocol. The Tanimoto similarity index (TSI) values computed between experimental and simulated VCD spectra are also given.

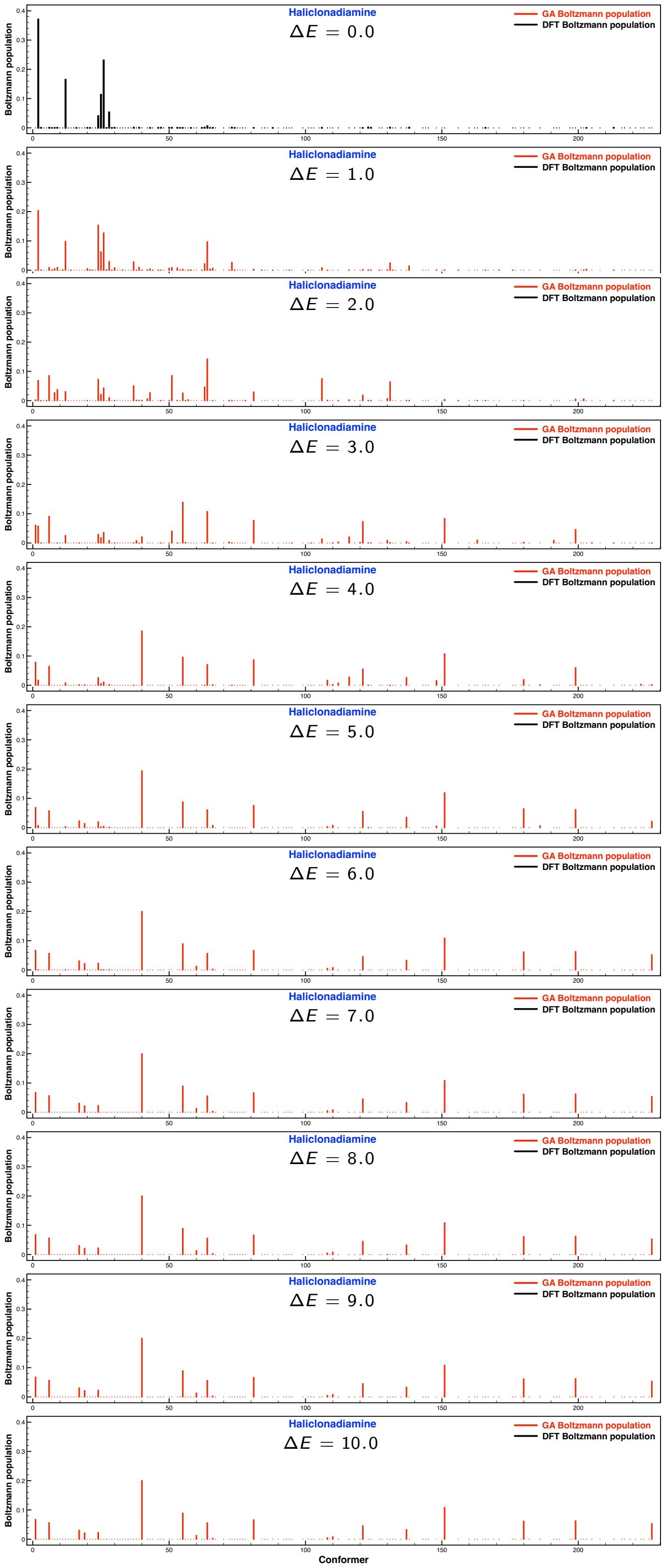

Figure S7: Dependence of the GA Boltzmann factors on the ΔE threshold for the relative energy uncertainty.

Papuamine:

1st three lowest-energy conformers predicted with DFT:

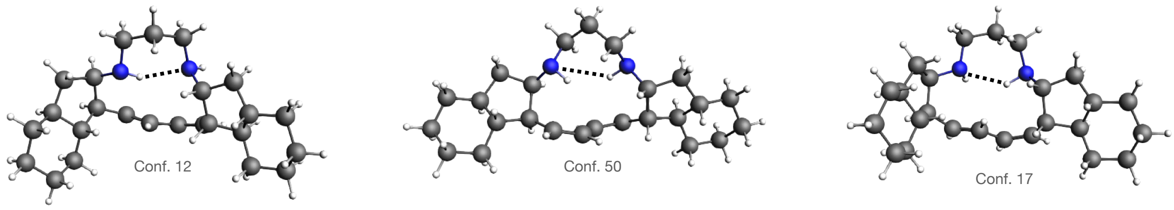

1st three lowest-energy conformers predicted by GA-VCD:

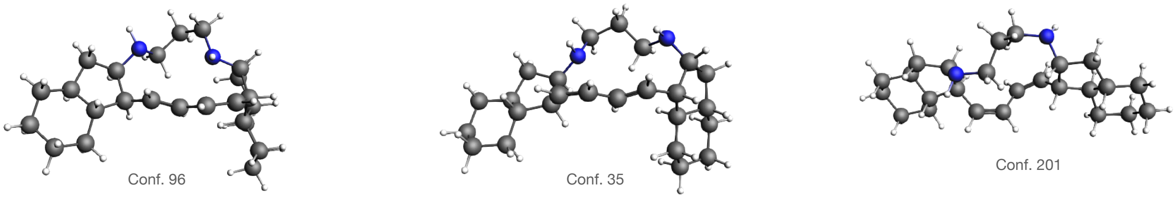

Haliclonadiamine:

1st three lowest-energy conformers predicted with DFT:

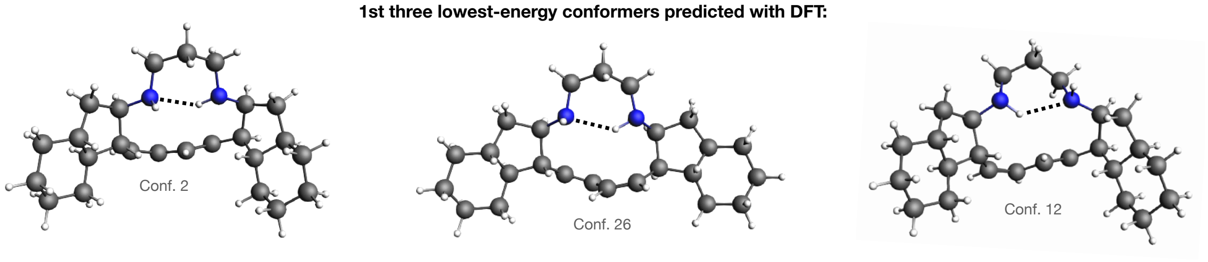

1st three lowest-energy conformers predicted by GA-VCD:

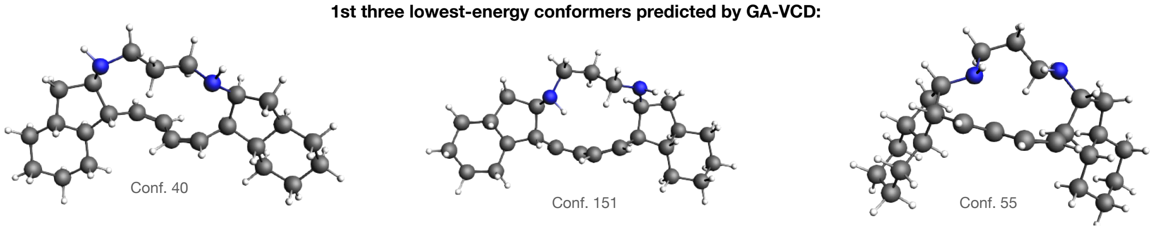

Figure S8: Structures of the haliclonadiamine and papuamine conformers which have been predicted by DFT and by the GA-VCD protocol to have the lowest energies.

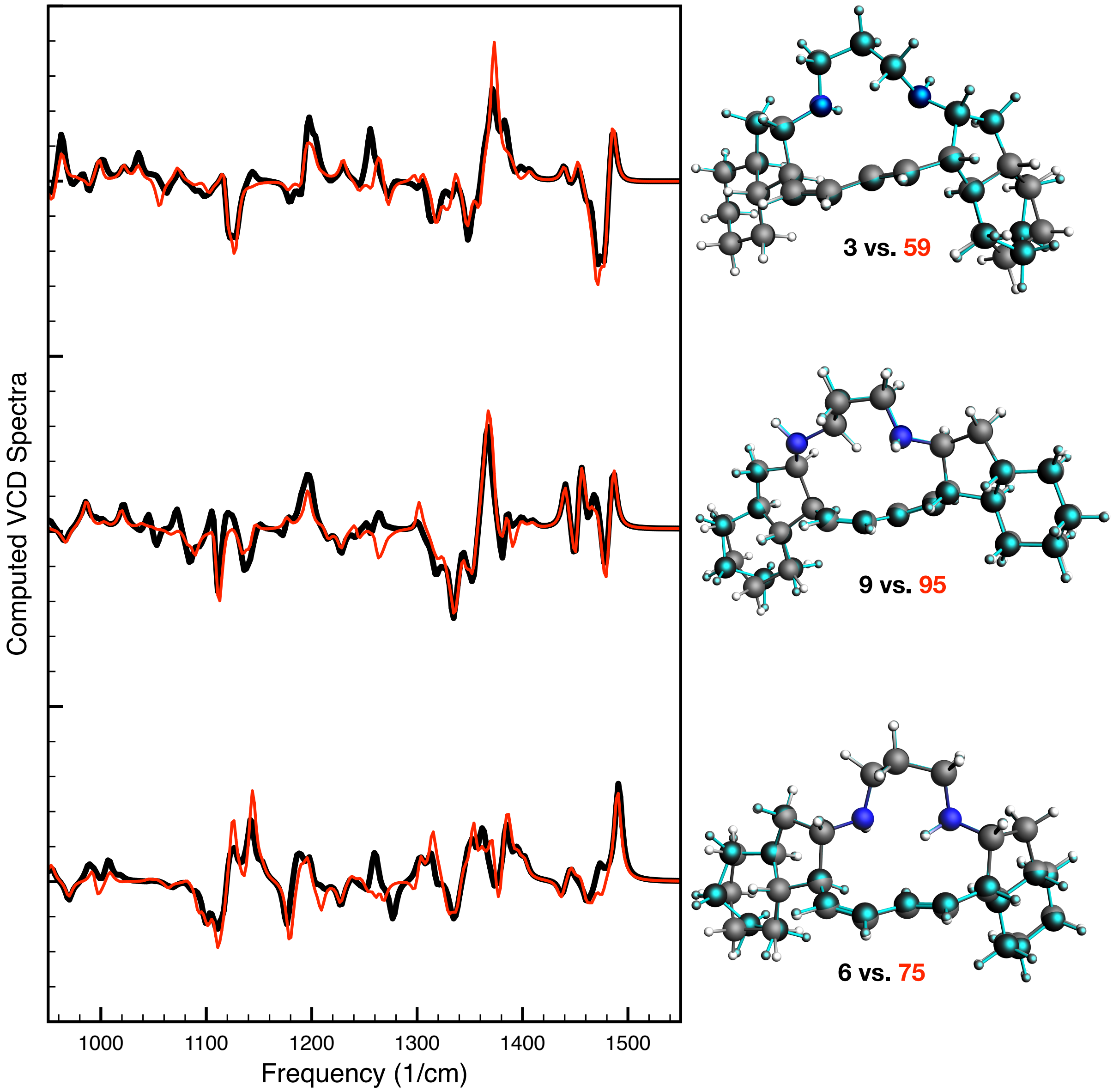

Figure S9: Structures and VCD spectra of the Haliclonadiamine conformers of three (randomly chosen) VCD families (see VCD dendrogram in Figure 7 in the main manuscript). Note that the only difference between the structures of the conformers in a given VCD families is a flip in one of the cyclohexane rings.

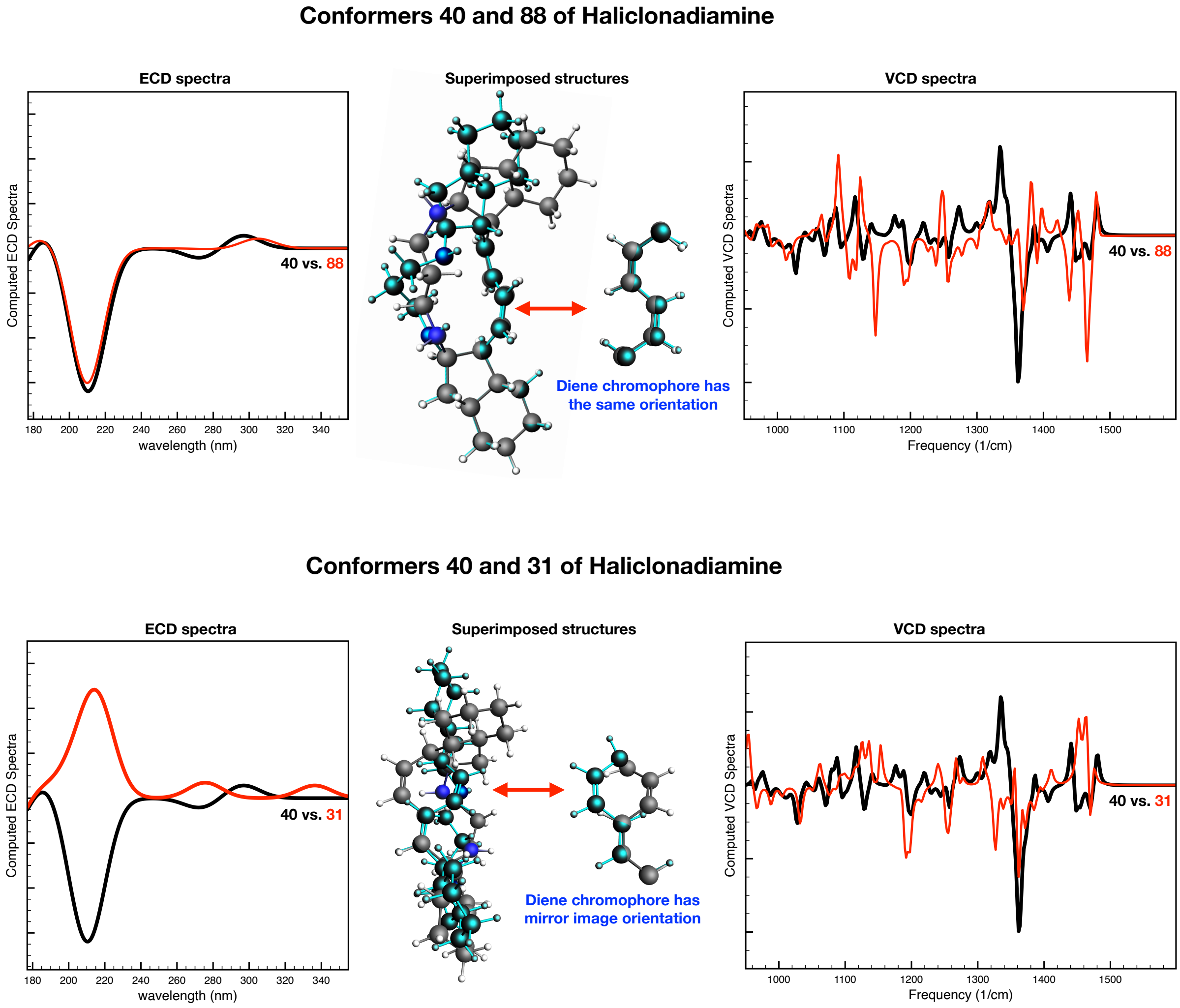

Figure S10: Sensitivity of the ECD vs. VCD spectra to differences in the molecular structure. Upper panel: even though the conformers 40 and 88 have different structures, the diene chromophore has the same conformation in these two structures. As a result, the associated ECD spectra are very similar. Lower panel: the diene chromophore has mirror-image orientation in conformers 40 and 31. As a result, the ECD spectra of these two conformers have almost perfect opposite sign. The VCD spectra of conformers 40, 88 and 31 are different.

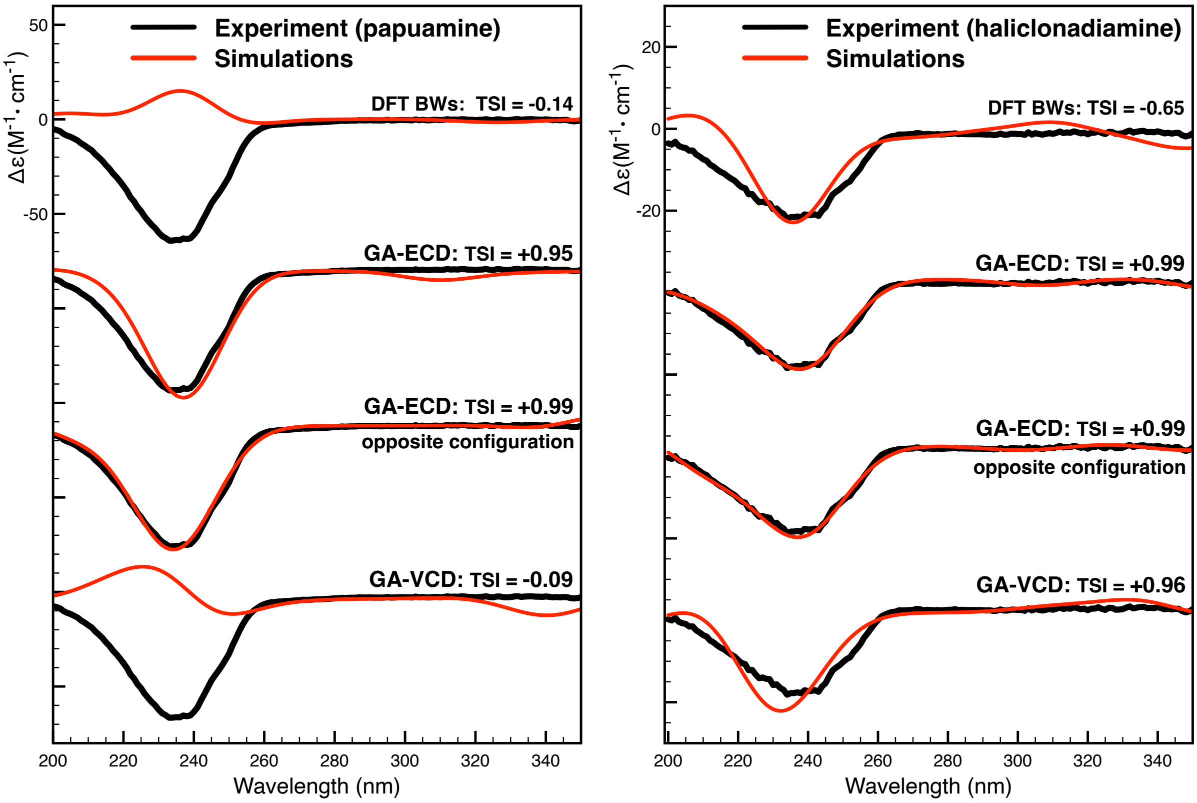

Figure S11: Application of the GA protocol to the papuamine and haliclonadiamine ECD spectra. As expected, the genetic algorithm can reproduce the experimental ECD spectra irrespectively of the conformer used in calculations. The spectra labeled "GA-VCD" have been obtained by averaging the ECD spectra using the Boltzmann factors obtained by applying the GA protocol to VCD spectra. It is important to note that the ECD and VCD spectra are typically measured in different solvents and as such the conformers populated in the VCD experiment may not be relevant in the ECD experiment, and vice versa. The Tanimoto similarity index (TSI) values computed between experimental and simulated spectra are also given.

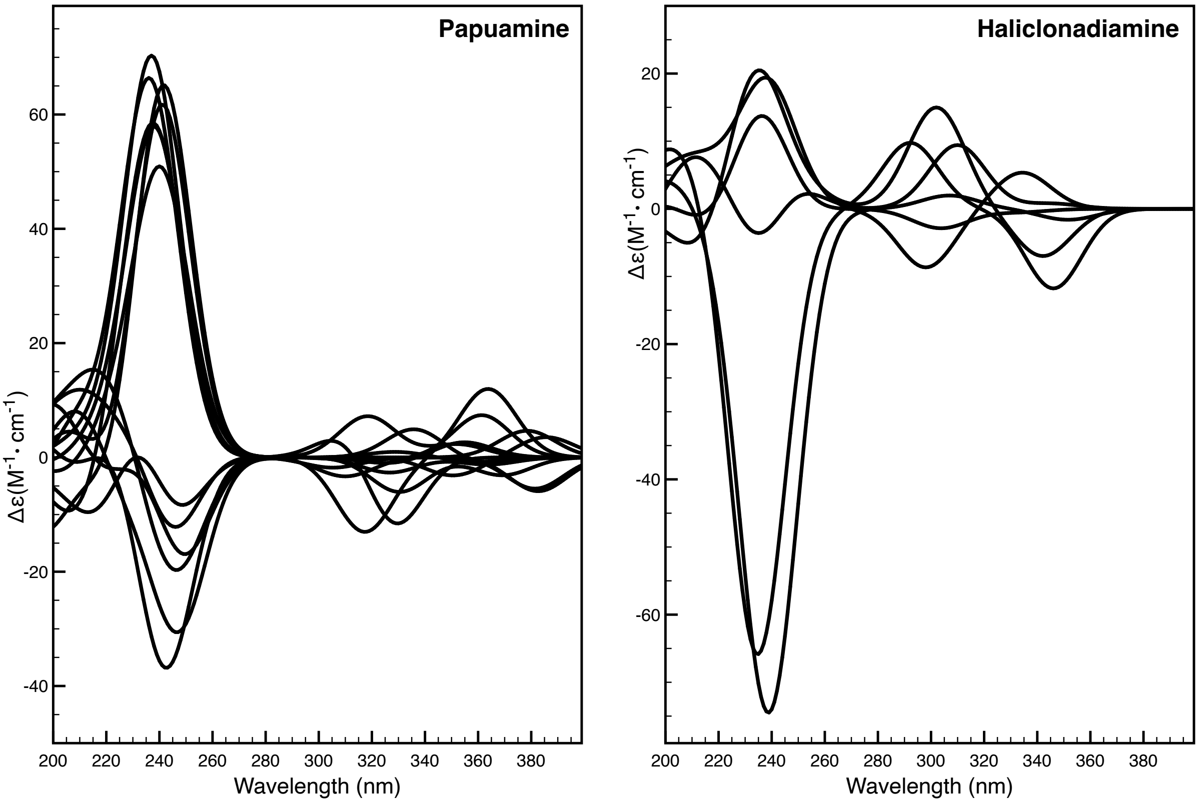

Figure S12: ECD spectra computed for the "DFT" conformers within an energetic window of 2.0 kcal/mol. Depending on the considere conformer, we have a positive or a negative band around 240 nm. Therefore, the sign of the simulated ECD spectrum at ∼240 nm (where the only experimental ECD band is situated), depends sensitively on the computed Boltzmann factor, which are inaccurate

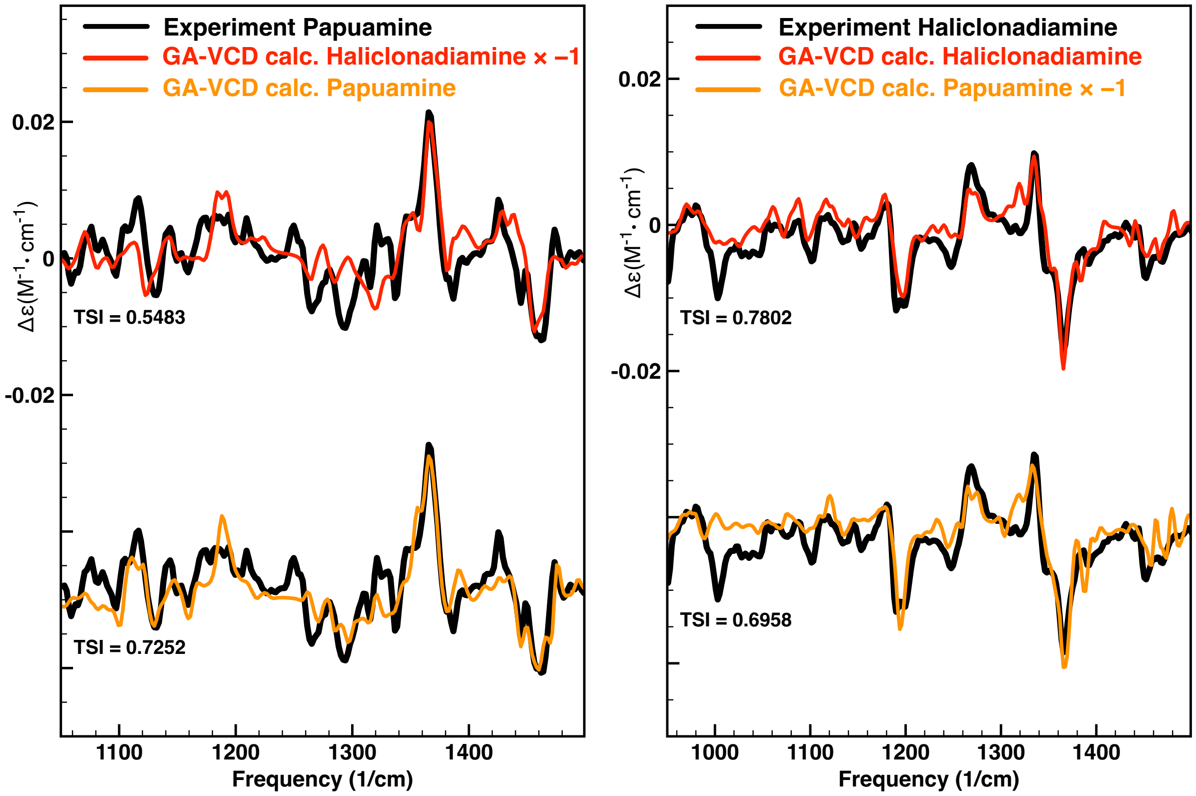

Figure S13: Diastereomer discrimination using the GA-VCD protocol. For more details, see Table IV in the main manuscript and the associated discussion. The Tanimoto similarity index (TSI) values computed between experimental and simulated spectra are also given.

Conf.	DFT-BWs	TSI	ΔE	H-bond
12	0.36	0.06	0.00	yes
50	0.14	0.03	0.55	yes
17	0.09	0.31	0.82	yes
6	0.08	0.29	0.91	yes
53	0.05	0.01	1.18	no
4	0.04	0.26	1.33	no
29	0.04	−0.05	1.33	no
11	0.03	0.04	1.56	yes
20	0.03	0.13	1.57	yes
2	0.02	0.29	1.61	yes
34	0.02	0.14	1.74	no
52	0.02	−0.10	1.80	yes
62	0.01	0.13	1.90	yes
21	0.01	0.05	2.02	yes
124	0.01	0.07	2.03	yes
Total:	0.95			

Conf.	GA-BWs	TSI	ΔE	H-bond
96	0.22	0.39	7.37	no
35	0.15	0.47	4.07	no
201	0.13	0.20	8.64	no
150	0.09	0.31	6.83	no
22	0.07	0.46	4.61	no
99	0.06	0.19	7.50	yes
119	0.04	0.20	4.92	yes
101	0.04	0.36	8.89	yes
65	0.03	0.31	7.32	no
72	0.03	0.34	8.77	yes
155	0.03	0.46	9.48	no
115	0.02	0.36	8.80	no
141	0.02	0.29	9.55	yes
193	0.02	0.29	9.85	no
100	0.02	0.42	9.91	yes
Total:	0.97			

Table S-VII: Tanimoto similarity indices (TSI) and Boltzmann weights (BW) of the most relevant papuamine conformers: predictions made by DFT (upper section) vs. predictions made by the GA-VCD protocol (lower section). All relative energies (ΔE) are computed with respect to conformer 2 (i.e., the lowest energy according to DFT) using the DFT energies. Relative energies are given in kcal/mol. The Tanimoto indices give the similarity between the experimental VCD spectrum and the DFT VCD spectra of the individual conformers. The 5th column indicates whether or not a given conformer exhibits a N–H···N–H intramolecular hydrogen bond.
